# Quantification of Lysine Acetylation and Succinylation Stoichiometry in Proteins Using Mass Spectrometric Data-Independent Acquisitions (SWATH)

**DOI:** 10.1007/s13361-016-1476-z

**Published:** 2016-09-02

**Authors:** Jesse G. Meyer, Alexandria K. D’Souza, Dylan J. Sorensen, Matthew J. Rardin, Alan J. Wolfe, Bradford W. Gibson, Birgit Schilling

**Affiliations:** 1 Buck Institute for Research on Aging, Novato, CA 94945 USA; 2 Amgen, South San Francisco, CA 94080 USA; 3 Department of Microbiology and Immunology, Stritch School of Medicine, Health Sciences Division, Loyola University Chicago, Maywood, IL 60153 USA; 4 Department of Pharmaceutical Chemistry, University of California, San Francisco, CA 94143 USA

**Keywords:** Stoichiometry, Acetylation, Succinylation, SWATH, Data-independent acquisition, Mass spectrometry, Skyline

## Abstract

Post-translational modification of lysine residues by N_Ɛ_-acylation is an important regulator of protein function. Many large-scale protein acylation studies have assessed relative changes of lysine acylation sites after antibody enrichment using mass spectrometry-based proteomics. Although relative acylation fold-changes are important, this does not reveal site occupancy, or stoichiometry, of individual modification sites, which is critical to understand functional consequences. Recently, methods for determining lysine acetylation stoichiometry have been proposed based on ratiometric analysis of endogenous levels to those introduced after quantitative per-acetylation of proteins using stable isotope-labeled acetic anhydride. However, in our hands, we find that these methods can overestimate acetylation stoichiometries because of signal interferences when endogenous levels of acylation are very low, which is especially problematic when using MS1 scans for quantification. In this study, we sought to improve the accuracy of determining acylation stoichiometry using data-independent acquisition (DIA). Specifically, we use SWATH acquisition to comprehensively collect both precursor and fragment ion intensity data. The use of fragment ions for stoichiometry quantification not only reduces interferences but also allows for determination of site-level stoichiometry from peptides with multiple lysine residues. We also demonstrate the novel extension of this method to measurements of succinylation stoichiometry using deuterium-labeled succinic anhydride. Proof of principle SWATH acquisition studies were first performed using bovine serum albumin for both acetylation and succinylation occupancy measurements, followed by the analysis of more complex samples of *E. coli* cell lysates. Although overall site occupancy was low (<1%), some proteins contained lysines with relatively high acetylation occupancy.

**Electronic supplementary material:**

The online version of this article (doi:10.1007/s13361-016-1476-z) contains supplementary material, which is available to authorized users.

**Graphical Abstract:**
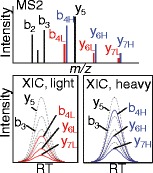
ᅟ

## Introduction

Post-translational modification of lysine residues by N_Ɛ_-acetylation can modulate protein activities, conformation, and protein–protein interactions [1, 2]. Lysine acetylation and other acylations are thought to play key roles in metabolism and cell signaling [3]. In eukaryotes, the acetylation and deacetylation of lysine residues in histones has long been recognized as a mechanism to regulate their binding to DNA [4, 5]. Recently, other proteins have been found to undergo acetylation, with a large number localized to the mitochondria [6, 7]. In addition to lysine acetylation, other types of lysine acylation modifications, including succinylation, malonylation, and glutarylation, are highly prevalent in mitochondrial proteins isolated from various mouse tissues and human cell lines [8–14]. Other studies have also found acylation modifications in bacteria, and proteins involved in central metabolism, translation, and transcription are heavily modified in *E. coli* [15–24]. For example, Colak et al. [21] identified 2803 lysine acetylation sites in 782 proteins and 2580 lysine succinylation sites in 670 proteins in wild-type *E. coli* strains.

In mitochondria, no lysine acyltransferase has been identified, leading to speculation that mitochondrial acylation may result from the nonenzymatic reaction of lysine with reactive acyl-CoAs [25, 26]. The removal of these modifications is regulated by mitochondrial NAD^+^-dependent deacylases, SIRT3 and SIRT5 [27–29]; SIRT3 is highly expressed in mitochondria-rich tissues, and expression in liver, heart, and skeletal muscle is differentially regulated in response to changes in nutrient availability [30, 31]. Several mass spectrometric studies have investigated SIRT3 knockout mice, which feature hyperacetylation of mitochondrial proteins [8, 9], and similar studies of SIRT5 knockout mice identified SIRT5-regulated sites of succinyl- and malonyl-protein modifications [10–13]. SIRT5 was also described as a de-glutarylase [14]. In bacteria, effects of lysine acetylation on central metabolism were first described for *E. coli* [32, 33]. Since then, several reports have described extensive remodeling of the *E. coli* acetylome in mutant strains lacking the sirtuin homolog CobB, [15–17, 21, 22, 34] or in response to certain nutrient conditions [22–24]. Recently, Svinkina et al. published an in-depth mass spectrometric study with an optimized acetylation enrichment workflow using a new monoclonal antibody mixture [35].

Although many studies have clearly shown that lysine acyl modifications undergo large fold-changes under different conditions, precise measures of acylation site occupancy, or stoichiometry, are scarce. Some investigators have used methods originally developed for estimating occupancy of protein phosphorylation [36] in stable-isotope labeling with amino acids in cell culture (SILAC) experiments by comparing peptide ion measurements obtained after affinity enrichment to their unmodified counterparts observed prior to enrichment [37]. However, these indirect methods can be problematic as they rely on several assumptions, for example, that the modified and unmodified peptide show similar ionization efficiency, which is usually not the case. Additionally, this method assumes that measured peptides are only modified by the modification of interest, which may be true in the case of phosphorylation but is often not true for lysine acylation; many distinct lysine modifications occur at the same position assessments. Using this strategy, Weinert et al. reported very low acetylation occupancy for SIRT3-targeted sites from mouse liver, where the vast majority of sites (97%) were <1% acetylated [38].

Recently, two groups have reported more direct methods for determining lysine acetylation site occupancies that determine the ratio of abundance of endogenous “light” acetyl groups to stable isotope-labeled “heavy” acetyl groups, the latter being generated by quantitative per-acetylation of unmodified lysines in vitro. For example, Nakayasu et al. labeled peptides with ^13^C_2_-acetic anhydride after Arg-C digestion followed by MS2-based quantification of acetyl lysine immonium ions [39], while Baeza et al. labeled intact proteins with acetic anhydride-*d*_*6*_ followed by trypsin digestion and precursor ion quantification of acetyl lysine-containing peptides pairs [15]. Using this approach, Baeza et al. made a comprehensive assessment of lysine acetylation stoichiometries in 899 *E. coli* proteins where lysine acetyl occupancy ranged from less than 1% to 98%. Stoichiometry was determined as 0–10% for ~2500 peptides, 10–20% for ~500 peptides, 20–30% for ~60 peptides, and greater than 30% for ~70 peptides. However, using this strategy, we found that stoichiometry measurements based on isotope ratios obtained from MS1 spectra are prone to overestimation because of signal interferences, particularly as most acetylation occupancies are very low (<1%). For example, accurate measurement of 1% acetylation occupancy requires an interference-free peptide signal over at least two orders of magnitude. Therefore, these experiments require both the determination of accurate isotopic abundances over a large dynamic range, and the ability to remove signal interferences that may otherwise distort these measurements. This is in contrast with a conceptually similar, commonly-used quantification method, SILAC, where the expected (null hypothesis) ratio is 1:1, and ratios greater than 10-fold are uncommon.

Quite recently, Zhou et al. [40] described an approach to assess lysine acetylation site stoichiometries that also employed quantitative acetylation of all free lysines in proteins with stable isotope labeling coupled to analysis using MS2 fragment ion intensities. While measuring ion intensity differences of MS2 ions likely improves overall accuracy by reducing interferences, quantification from data-dependent acquisition (DDA) poses other problems. For example, using DDA-generated MS/MS spectra for quantification can suffer from stochastic sampling deficiencies, as some precursors may never be selected for MS/MS due to their low abundances. Another issue is that light and heavy peptide precursors may be selected for MS/MS at different points during peak elution, resulting in distorted ratios, especially with deuterated species that can elute slightly before their protonated counterparts [41].

To address the challenges inherent in accurate quantification of lysine acylation stoichiometry, we have developed a method that applies a variation of the stable isotope labeling method from Baeza et al. [15], followed by SWATH acquisition that collects both precursor and multiple fragment ion abundances [42]. We first benchmarked this new acylation occupancy workflow with experiments using (acetylated) bovine serum albumin (BSA), and then we investigated protein lysates from *E. coli* under different growth conditions where we had previously observed large fold-changes in acetylation levels [23], but where data on the site occupancy of these changes was not known. We also present a tractable method for data analysis using a combination of Skyline and custom scripts written in-house. The combination of our SWATH fragment ion quantification and our custom software allow us to determine site-specific acylation stoichiometry from peptides containing multiple lysines. We extended the method to allow determination of lysine succinylation stoichiometry. Our results indicate that using SWATH fragment ion quantification improves accuracy and precision compared to results from only precursor quantification. We find the majority of both lysine acetylation and succinylation site occupancies in *E. coli* are relatively low (<1%–5%), but some sites have higher stoichiometry (>5%).

## Experimental

### Chemicals

Acetonitrile and water were obtained from Burdick and Jackson (Muskegon, MI, USA). Reagents for protein chemistry including iodoacetamide, dithiothreitol (DTT), ammonium bicarbonate, formic acid (FA), urea, succinic anhydride, acetic anhydride-*d*_*6*_ (>99% deuterium atom enrichment), and succinic anhydride-2,2′,3,3′-*d*_*4*_ (>98% deuterium atom enrichment) were purchased from Sigma Aldrich (St. Louis, MO, USA). BSA and acetylated BSA were purchased from Pierce (Rockford, IL, USA). Sequencing grade Glu-C endoproteinase was purchased from Roche (Indianapolis, IN, USA). HLB Oasis SPE cartridges were purchased from Waters (Milford, MA, USA).

### K-acyl Enrichment from Digested E. coli Lysate

Wild-type *E. coli* K-12 strain BW25113 was grown in TB7 (which contains 1% wt/vol tryptone buffered at pH 7.0 with 100 mM potassium phosphate) either supplemented or not with 0.4% glucose [23]; alternatively, for some experiments, TB7 was supplemented with 33 mM succinic acid. Cell pellets were collected and lysed as previously described by Schilling et al. [23]. Briefly, proteins were resuspended in 6 M urea, 100 mM Tris, 75 mM NaCl containing the deacetylase inhibitors 1 mM tricostatin A, and 3 mM nicotinamide. Typically 1.5 mg of protein lysate was reduced with 20 mM DTT (37 °C for 1 h), and alkylated with 40 mM iodoacetamide (30 min at RT in the dark). Samples were diluted 10-fold with 100 mM Tris pH 8.0 and incubated overnight at 37 °C with sequencing grade Glu-C (Roche) added at a 1:50 enzyme:substrate ratio (wt:wt). Samples were acidified with formic acid and desalted using HLB Oasis SPE cartridges (Waters) [43]. Proteolytic peptides were eluted, concentrated to near dryness, and resuspended in NET buffer (50 mM Tris-HCl, pH 8.0, 100 mM NaCl, 1 mM EDTA). Proteolytic acetyllysine-containing peptides were affinity-purified using the agarose conjugated polyclonal anti-acetyllysine antibody from ImmuneChem (ICP0380-100) that was prewashed and incubated with 1 mg digested *E. coli* protein lysate (in NET buffer) overnight (4 °C) at a 1:25 antibody:peptide ratio (wt:wt) as previously described [23]. Prior to mass spectrometric analysis, the acetyllysine peptide enrichment fractions were concentrated and desalted using C-18 zip-tips (Millipore, Billerica, MA, USA). Similarly, K-succinyl peptides were enriched (for identification purposes) from 1 mg of Glu-C-digested *E. coli* protein lysate using the PTMScan agarose conjugated anti-succinyllysine antibody from CST, a 7 monoclonal Ab mixture, according to the manufacturer’s protocol except that 1/4 of an aliquot (20 μL slurry) of antibody-bead conjugate was used per 1 mg of digested *E. coli* protein lysate. Prior to mass spectrometric analysis, the enriched peptides were concentrated and desalted using StageTips made in house with three disks of Empore C18 material (3 M, Minneapolis, MN, USA). Acetylated and succinylated peptides and proteins that were identified by mass spectrometric analysis from these enrichment protocols are listed in Supplementary Table S1a–c.

### Chemical Treatments to Quantitatively Acetylate and Succinylate Proteins—Acylation Stoichiometry

BSA Per-Acetylation: 100 μg of BSA protein was diluted to 1 μg/uL using ammonium bicarbonate solution (8 M urea, 200 mM ammonium bicarbonate pH 8). Dithiothreitol was added to a final concentration of 20 mM and incubated at 37 °C for 30 min. Iodoacetamide was added to a final concentration of 40 mM and incubated in the dark at room temperature for 30 min. Chemical per-acetylation was performed using an adjusted protocol from Baeza et al. [15] by adding 60 μmol of acetic anhydride*-d6* (Sigma Aldrich) to the sample and incubating in a Thermomixer at 4 °C for 20 min. The chemical acetylation process was repeated twice more. Ammonium hydroxide was added between each per-acetylation reaction to increase the pH to ~8, and 5 μL of 50% hydroxylamine was added after the final reaction to revert O-acetylation side reactions (note: the labeling reaction can also be carried out using 200 mM triethanolamine bicarbonate, TEAB, instead of ammonium bicarbonate). The urea concentration was diluted to 0.8 M using 25 mM ammonium bicarbonate and the protease Glu-C was added at a 1:50 ratio (wt:wt) and incubated overnight at 37 °C. Samples were then acidified by the addition of formic acid to 1% by volume and desalted using Oasis HLB extraction cartridges. The eluate was dried in a Speed-Vac and resuspended in 0.1% formic acid with 2% ACN. For per-acetylation of the *E. coli* lysate, 1 mg protein lysate was reacted with 60 μmol acetic anhydride-d_6_ per each of the three subsequent per-acylation incubations. Per-succinylation was performed using the same protocols as for the per-acetylation experiments described above (for BSA experiments and for *E. coli* lysate experiments, respectively). The succinic anhydride-*d*_*4*_ powder was dissolved in dry DMSO prior to the reaction and used at the same molarity as described above for the acetic anhydride*-d*_*6*_.

### Offline Peptide Fractionation by Basic-pH Reversed Phase HPLC

Per-acylated and Glu-C digested proteins from whole *E. coli* lysates were separated by a Waters 1525 binary HPLC pump system including a Waters 2487 UV detector. The high-pH reversed phase peptide separation was performed using an Agilent Zorbax 300Extend C18 column (4.6 mm × 250 mm, 5 μm particle size; Agilent, Santa Clara, CA, USA). Peptides were separated at a flowrate of 0.7 mL/min using the following gradient: 100% A to 92% A over 7.3 min, 92% A to 73% A over 38 min, 73% A to 69% A over 4 min, 69% A to 61% A over 16 min, 61% A to 40% A over 7 min, 40% A to 10% A over 7.73 min, constant 10% A from 80 to 85 min, 10% A to 100% from 85 to 86 min, and finally re-equilibrated in 100% A for 24 min. Buffer A was 10 mM ammonium formate in water, and buffer B was 10 mM ammonium formate in 90% ACN and 10% water. The pH of both mobile phases adjusted to 10 with neat ammonia. Fractions were automatically collected and every eighth fraction was pooled, resulting in eight final fractions as described previously [35, 44].

### Mass Spectrometry

Samples were analyzed by reverse-phase HPLC-ESI-MS/MS using the Eksigent Ultra Plus nano-LC 2D HPLC system (Dublin, CA, USA) combined with a cHiPLC System, which was directly connected to a quadrupole time-of-flight SCIEX TripleTOF 5600 or a TripleTOF 6600 mass spectrometer (SCIEX, Redwood City, CA, USA). Typically, mass resolution in precursor scans was ~35,000 (TripleTOF 5600) or 45,000 (TripleTOF 6600), whereas fragment ion resolution was ~15,000 in ‘high sensitivity’ product ion scan mode. After injection, peptide mixtures were transferred onto a C18 pre-column chip (200 μm × 6 mm ChromXP C18-CL chip, 3 μm, 300 Å, SCIEX) and washed at 2 μL/min for 10 min with the loading solvent (H_2_O/0.1% formic acid) for desalting. Subsequently, peptides were transferred to the 75 μm × 15 cm ChromXP C18-CL chip, 3 μm, 300 Å, (SCIEX), and eluted at a flow rate of 300 nL/min using a 2 h gradient using aqueous and acetonitrile solvent buffers.

Some initial data-dependent acquisitions (DDA) were carried out to obtain MS/MS spectra for the 30 most abundant precursor ions (100 ms per MS/MS) following each survey MS1 scan (250 ms), yielding a total cycle time of 3.3 s as previously described [22, 45]. For collision induced dissociation tandem mass spectrometry (CID-MS/MS), the mass window for precursor ion selection of the quadrupole mass analyzer was set to ±1 *m/z* using the Analyst 1.7 (build 96) software. All stoichiometry study samples were analyzed by data-independent acquisitions (DIA), or specifically variable window SWATH acquisitions. In these SWATH acquisitions, instead of the Q1 quadrupole transmitting a narrow mass range through to the collision cell, windows of variable width (5 to 90 *m/z*) are passed in incremental steps over the full mass range (*m/z* 400–1250). The cycle time of 3.2 s includes a 250 ms precursor ion scan followed by 45 ms accumulation time for each of the 64 SWATH segments. The variable windows were determined according to the complexity of the typical MS1 ion current observed within a certain *m/z* range using a SCIEX ‘variable window calculator’ algorithm (i.e., more narrow windows were chosen in ‘busy’ *m/z* ranges, wide windows in *m/z* ranges with few eluting precursor ions). In addition, selected samples were acquired in parallel reaction monitoring (PRM) mode, monitoring light and heavy pairs of specific peptides as previously described by Schilling et al. [46].

### Data Processing and Bioinformatics

Mass spectrometric wiff files were first converted to mzML using the SCIEX data converter version 1.3, and subsequently mzML files were converted to mzXML files using ProteoWizard version 3.0.8851. The DIA-Umpire [47] signal extraction module was used to process SWATH acquisitions, which detects correlated precursor and fragment ion features and assembles them into pseudo-tandem MS/MS spectra stored in mgf files (for details on settings and parameters see Supplementary Table S2). These pseudo MS/MS spectra derived from all SWATH acquisitions were then searched using the database search engine Mascot [48] server version 2.3.02. In addition, DIA-Umpire-derived mgf files containing pseudo MS/MS spectra were searched with MSGF+ [49], refined with PeptideProphet [50], and combined with iProphet [51]. For the database searches, enzymatic cleavage sites were defined at Glu (E) and Asp (D), and fixed modifications were set as carbamidomethylation for cysteine residues. Variable modifications were defined for lysine residues as light and heavy acetyl for acetylation occupancy experiments and as light and heavy succinyl for succinylation occupancy experiments. False discovery rates (FDR) were required to be <0.01. The search engine results were subsequently used in Skyline to build spectral libraries for SWATH data processing in Skyline.

SWATH acquisitions were quantitatively processed using Skyline 3.5 [52], an open source software project (http://proteome.gs.washington.edu/software/skyline). Quantitative MS1 analysis (from SWATH acquisitions) was based on extracted ion chromatograms (XICs) for the top three resulting precursor ion peak areas (e.g., M, M + 1, and M + 2 [42, 45]. Final quantitative comparisons were typically based on only the highest ranked precursor ion. Quantitative SWATH MS2 data analysis was based on extracted ion chromatograms (XICs) of up to 10 of the most abundant fragment ions in the identified spectra, but in contrast to MS1 quantification, only ions containing the modified lysine of interest were used for quantification, as those can be used to differentiate between the light and heavy acylated peptide forms. Occupancy measurements were computed from light (L) and heavy (H) peak areas as L/(L + H).

For peptides containing only one lysine, Python (version 2.7.10) scripts written in-house were used to determine the differentiating fragment ions and compute the stoichiometry from custom Skyline reports. To enable quantification of peptides containing more than one lysine modification, an additional R script (http://www.R-project.org/), referred to as StoichiolyzeR (https://github.com/GibsonLab/StoichiolyzeR), was written in-house and uses the package mzR [53] to extract fragment ion signal from the mzXML file and to compute the stoichiometry of acylation sites. Briefly, the StoichiolyzeR program requires an mzXML file generated from the original wiff raw data file, a text file containing the SWATH isolation window definitions, and a text file containing peptide information, such as a Skyline report. The peptide information text file contains the peptides sequence, peak retention time borders for peak area extraction/integration, precursor charge, mzXML file name, and the peptide sequence residue numbers to define the K-acyl sites within the protein. The program first determines all theoretical heavy and light fragment ion *m/z* values that contain the lysine residue(s). The program also confirms that the heavy/light precursor ion pairs were sampled in the same SWATH isolation window. Next, the heavy and light fragment peak areas are extracted using a predefined ppm mass tolerance within the given retention time elution range. Fragment ion peak areas subsequently were filtered by a user-defined minimum heavy peak area to avoid reporting very noisy, low abundant ions; however, if heavy peak areas are below the set threshold, the same threshold is tested for the corresponding light fragment ion (in case a specific K-acyl site shows a very high stoichiometry in which case heavy peak areas could be very low). Fragment ions are further processed if either the heavy or the light peak area is above the threshold. The ratios of light/(light + heavy) are computed, and both the median ratio and the ratio from the highest ranked (differentiating) fragment ion (rank 1) are reported. The stoichiometry for peptides containing two lysine residues per peptide is computed for the first and second lysine using the differentiating b-ion and y-ion ratios, respectively.

### Data Accession

The supplementary files provide further insights into the SWATH stoichiometry workflows, and advantages of using SWATH acquisitions to determine acylation occupancy. All raw files are uploaded at to the Center for Computational Mass Spectrometry, MassIVE (massive.ucsd.edu), and can be accessed using the MassIVE ID number: MSV000079779 (ProteomeXchange Accession PXD004234).

## Results and Discussions

### Acetyl and Succinyl Stoichiometry Workflows

**Figure 1:**
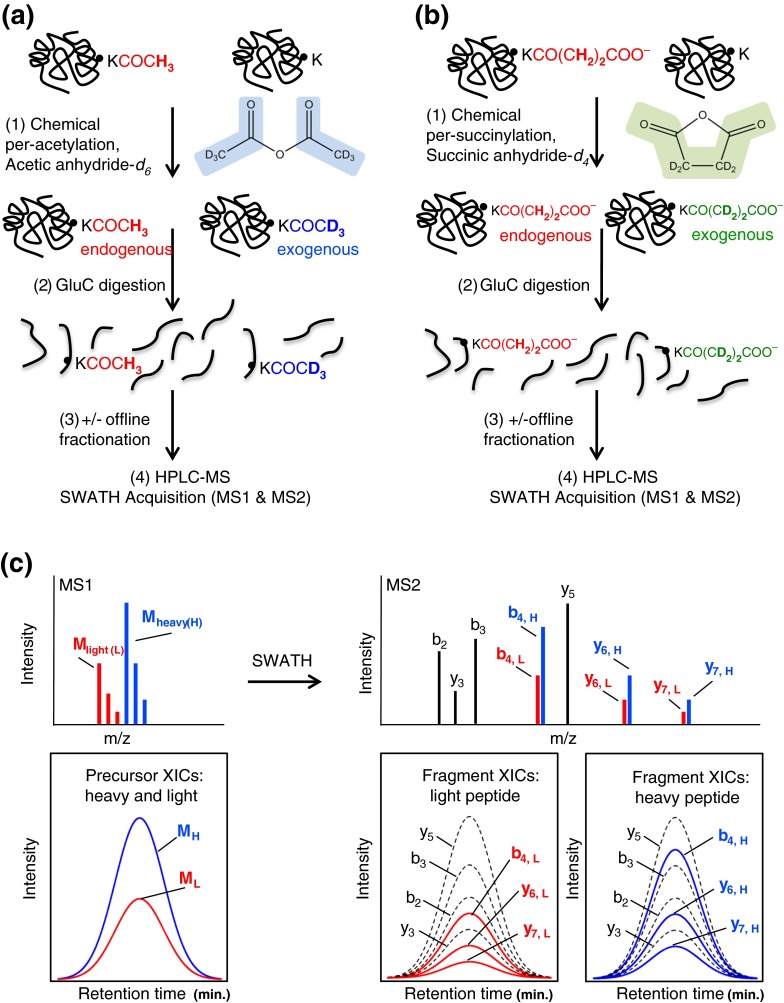
Stoichiometry workflow. **(a)** First, protein lysates were incubated three times with acetic anhydride-*d6* to acetylate unmodified lysine residues. Second, samples were digested with endoproteinase Glu-C, followed by optional offline fractionation of the proteolytic peptides by basic reversed-phase chromatography. Finally, peptides were analyzed by LC-MS in SWATH acquisition mode. **(b)** To determine succinylation stoichiometry the same workflow was used as described in **(a)**, except the heavy acylation reagent was changed to using succinic anhydride-*d4*. **(c)** Simulation of data obtained and stoichiometry calculations. Light and heavy precursor ions containing one acetylated lysine differ by 3 mass units. XICs were generated for both light and heavy isotopic envelopes indicated in red and blue, respectively (left panels). Similarly, quantification from fragment ion XICs from SWATH acquisitions can be performed using differentiating light and heavy fragment ions that contain the acetylation site indicated in red and blue (right panels). Common fragment ions are displayed in black and do not contain the site of modification

Our modified approach to determine acetylation stoichiometry was based on the method originally described by Baeza et al. [15], which depends on the accurate determination of isotopic peptide isoforms generated in vivo (endogenous) and in vitro (exogenous), the latter by per-acetylation of proteins with deuterated acetic anhydride-*d*_*6*_, followed by LC/MS and data analysis (Figure 1). Compared to their method, we altered several sample preparation steps. First, we increased the concentration of heavy acetic anhydride 3-fold to increase the chemical acylation efficiency for all three chemical per-acetylation reactions. Second, after the final acylation reaction, proteins were treated with hydroxylamine to more completely reverse O-acylation side reactions [39, 54]. Third, we digested the per-acetylated proteins with endoproteinase Glu-C instead of trypsin. Trypsin cannot cleave at C-terminal acylated lysine residues, and thus tryptic digestions of per-acylated proteins typically generate peptides that are very large and often contain many acetylated lysine residues, complicating analysis. Glu-C, however, is specific for C-terminal cleavage at glutamic and aspartic acid residues, and the cleavage pattern or efficiency of Glu-C should not be impacted by the presence of lysine acylation. In addition, we observed that peptides from Glu-C digestion are more likely to produce both b- and y-ion series due to the lack of a C-terminal basic residue, offering more flexibility during data analysis when choosing fragment ions that contain the acetyllysine for quantification. Therefore, we find that Glu-C digestion is preferable to trypsin digestion for per-acylated proteins. Finally, we have extended the lysine acetylation occupancy method to also allow measurements of succinylation occupancy (Figure 1b) by substituting the acylation reagent acetic anhydride*-d*_*6*_ with cyclic succinic anhydride-*d*_*4*_.

We also implemented two key post-digestion steps that differ from previous methods in order to further reduce quantification interferences compared with previous methods: peptide fractionation by offline basic pH reversed phase separation and quantification from fragment ions produced by SWATH acquisitions. Our SWATH method measures both the precursor peptide ion abundances and the fragment ion abundances. To quantify site occupancy, Skyline was used to extract peak areas from the fragment ions that contain the acylation site in question, hereafter referred to as ‘differentiating fragment ions’ (Figure 1c). Fragment ions that do not contain the modification have the same light and heavy *m/z* value and can be used for peptide identification but not for quantification. Typically, several differentiating *y-* and/or *b-*ions are measured that can be used for occupancy calculations, which provides flexibility if MS2-interferences were detected.

### SWATH MS2-Based Occupancy Workflow Assessment Using BSA Standards

**Figure 2:**
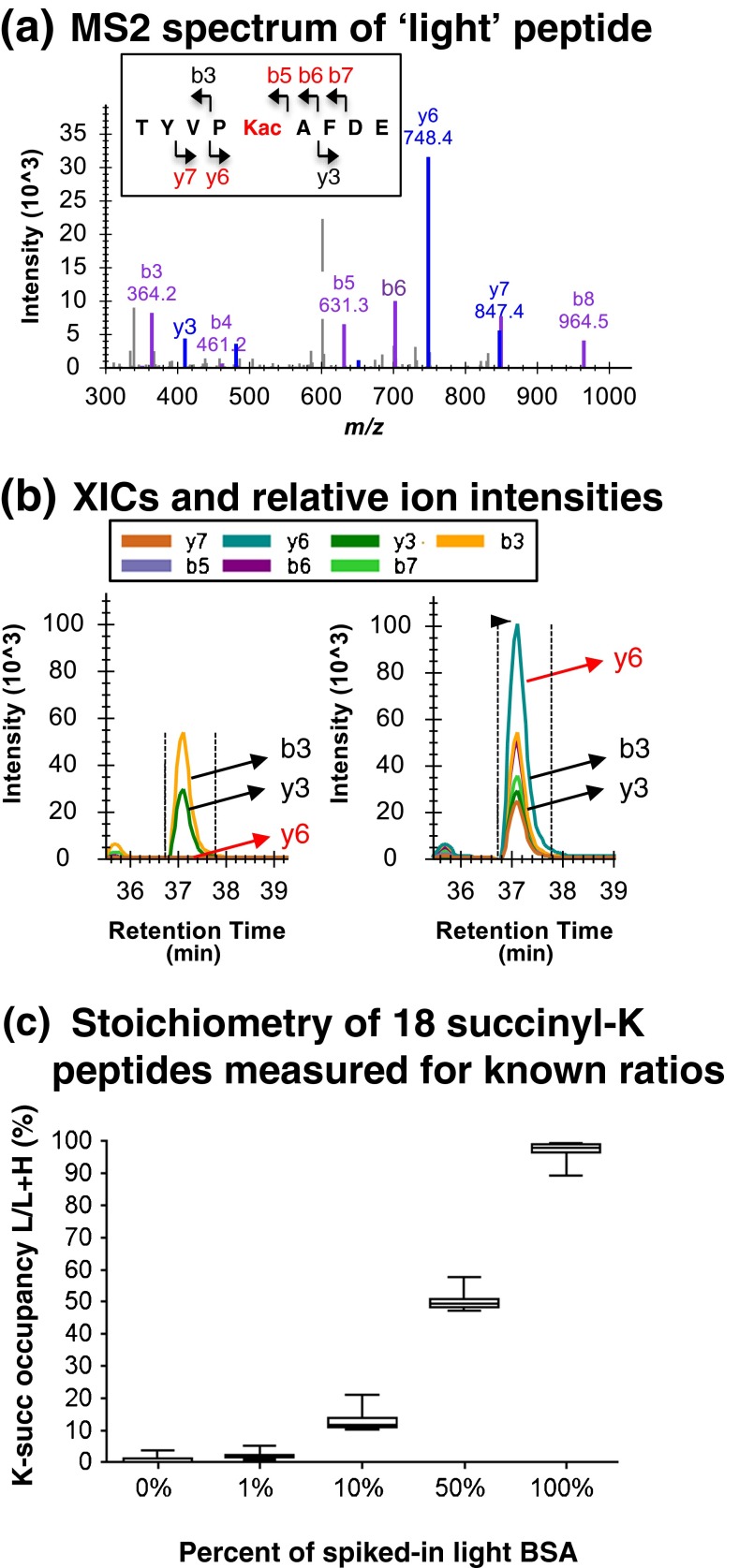
Assessing BSA acylation stoichiometry. **(a)** MS/MS spectrum for acetylated BSA peptide TYVP**Kac**AFDE displayed for the light precursor ion at 1110.54 Da with *m/z* 556.27. Differentiating ions are indicated in red in the fragmentation schematic (see inset). **(b)** Fragment ion XICs for light and heavy precursor ions at *m/z* 556.27 and *m/z* 557.78, respectively. The differentiating y_6_ ion is used for occupancy calculations. **(c)** Site occupancy for 18 succinylated BSA peptides were determined from the highest ranked differentiating fragment ions for five different BSA samples at defined percentages of light modification (e.g., at 0%, 1%, 10%, 50%, and 100%)

As a proof of principle demonstration of our modified DIA-based stoichiometry approach, we performed several experiments using commercially available acetylated and unmodified BSA. First, unmodified BSA was processed for stoichiometry assessments as shown in Figure 1. The efficiency of the chemical reaction was assessed by monitoring the disappearance of the unmodified species and the appearance of the corresponding N-acetylated species (N-acetyl-lysine). A potential by-product, the O-acetylated species, was barely detected at very low levels (~0.25% of N-acetylated main reaction product, Supplementary Figure S1), confirming the high specificity of the chemical per-acetylation reaction. Acetylated peptides identified by database searches were imported into Skyline with fragment ions ranked by the observed MS/MS spectra present in Skyline spectral libraries (Figure 2a and Supplementary Figure S2a). SWATH data was then imported into Skyline to extract precursor and fragment ion signals from the light and heavy acetyl-peptide pairs as shown in Figure 2b to quantify acylation occupancy. We typically use the highest ranked differentiating fragment ion per peptide based on spectral library ranking, and/or the second highest ranked differentiating fragment ion for occupancy calculations, as higher ranked ions typically have better ion statistics and less noise or interferences. This point is illustrated in Supplementary Figure S2b showing occupancy measurements for unmodified BSA, which is expected to have very low endogenous modification, where the L/(L + H) ratios were calculated for 18 peptides displaying measurements for multiple differentiating fragment ions per peptide, sorted by their MS/MS library rank; stoichiometry values calculated from fragment ions of ranks 1 and 2 are lower and more accurate compared with values calculated using less abundant fragment ions.

Corresponding calculations were also performed for the MS1 precursor ions sorted by their isotope distribution rank (Supplementary Figure S2b). It is immediately evident that even when analyzing a single, purified protein, MS1-based occupancy calculations (Supplementary Figure S2b-left panel) were incorrect, whereas the fragment ion-based calculations (Supplementary Figure S2b-right panel) were more accurate, showing acetylation occupancy values around 1% or less for endogenous, unmodified BSA (Supplementary Table S3a–d). A 95% CI) for MS2-based acetyl occupancy was determined to be between 0.54% and 1.29% with a mean of 0.82%. Additional measurements for commercially acetylated BSA sample are listed in Supplementary Table S3. All measurements were performed in three technical MS injection replicates, and technical reproducibility for occupancy per each of the peptides was robust with standard deviations between 0.03% and 0.76% using our new SWATH MS2-based quantification (see Supplementary Table S3).

After initially using manual annotation to indicate highest ranked differentiating fragment ions per K-acyl L/H peptide pair, we constructed a Python script to automatically determine the differentiating ions to use for occupancy calculations (see Methods and Supplementary Table S3). Overall, these proofs-of-principle experiments using acylated BSA standards showed that SWATH-MS2 based quantification of acylation occupancy provided accurate and precise measurements for both low and higher stoichiometries. These experiments also revealed that it is best to use rank 1 fragment ions for quantification as they typically show the best ion statistics and least interferences.

The methodology was extended to allow for succinylation stoichiometry determination by substituting succinic anhydride*-d*_*4*_ as the acylation reagent. We first validated the accuracy of this method using BSA to prepare defined levels of 1%, 10%, 50%, and 100% light succinylated BSA. Figure 2c shows SWATH MS2-based occupancy measurements of 18 BSA peptides at each of the predefined light succinylation levels. The measured occupancy readings matched the predefined levels very well (Figure 2c and Supplementary Table S3e). We measured succinylation occupancies for the predefined mixtures of 0%, 1%, 10%, 50%, and 100% light succinylation as 0.8%, 2.0%, 12.6%, 50.1%, and 97.3% with good precision (standard deviations were 1.0%, 1.0%, 2.6%, 2.5%, and 2.8%, respectively). These results are in accordance with the isotopic purity of the succinic anhydride-*d*_*4*_, which is reported at >98%. The isotopic purity for the acetic anhydride*-d*_*6*_ is reported at 99%. Incomplete isotopic enrichment of the labeling reagents will lead to a small overestimation of 2% (succinyl) or 1% (acetyl) of the endogenous acylation level; thus the determined occupancy levels are upper limit values.

### Acylation Stoichiometry in Complex E. coli Whole Lysate

**Figure 3:**
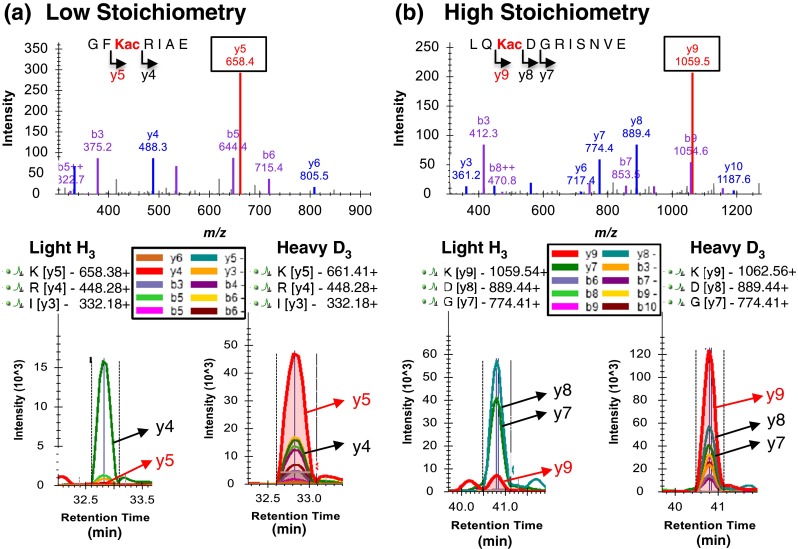
Lysine acetylation site occupancy examples from *E. coli* whole cell lysate. **(a)** A low stoichiometry of 0.5% was determined for acetylated peptide GF**Kac**RIAE representing K-105 in protein PTGA (P69783). The MS/MS is shown for the doubly charged light precursor ion at *m/z* 431.74. Fragment ion XICs derived from the corresponding light and heavy precursor ions at *m/z* 431.74 and *m/z* 433.25 are shown below. The differentiating y_5_ ion was used for occupancy calculations. **(b)** An example for a 5% stoichiometry was determined from acetylated peptide LQ**Kac**DGRISNVE for K-25 of protein LRP (P0ACJ0). The MS/MS for the light, doubly charged precursor ion at *m/z* 650.85 is shown, as well as fragment ion XICs for the corresponding light and heavy precursor ions at *m/z* 650.85 and *m/z* 652.36. The differentiating y_9_ ion was used for occupancy calculations

After validation of our stoichiometry quantification workflow with BSA standards, we applied the method to an *E. coli* whole protein lysate as shown in Figure 1. For this pilot experiment, we required that the lysine acylation site was previously identified independently by an anti-acyl enrichment analysis from Glu-C digestions (Supplementary Table S1a–c). Using this stringent set of confirmed acetylated peptides, we were able to manually inspect the peak areas in Skyline providing further validation of the method. Figure 3 shows example quantification of two *E. coli* acetylation sites, one with relatively high stoichiometry, and the other low. Specifically, Figure 3 shows how light/heavy differentiating fragment ions can be chosen for quantitative processing, and how the XICs appear in Skyline after fragment ion extraction. Acetylation site occupancy for K-105 of glucose-specific phosphotransferase enzyme IIA component (PTGA) was calculated from the fragment ion peak area ratio (Figure 3a) using the highest ranked differentiating ion, *y*_*5*_. While the heavy *y*_*5*_ ion signal was above 40,000 counts, the light version of the *y*_*5*_ ion was observed near baseline levels, yielding an estimated K-105 acetylation occupancy of only 0.5%. In comparison, a relatively high acetylation site occupancy of ~5% for K-25 of leucine-responsive regulatory protein (LRP) was determined using the *y*_*9*_ fragment ion peak areas (Figure 3b), which may be biologically relevant. Supplementary Table S4 shows an overview of many acetylation sites investigated for occupancy and, indeed, many lysine acetylation sites showed occupancy <1% when processing the SWATH data on the MS-based fragment ion level.

Another example of a relatively high level acetylation stoichiometry is shown in Supplementary Figure S3a for K-100 of 2,3-bisphosphoglycerate-dependent phosphoglycerate mutase (GPMA), which is further detailed in Supplementary Table S5. We previously showed GPMA acetyllysine site K-100 to be sensitive to acetyl phosphate [22] and glucose supplementation [23]. Indeed, with glucose added to the growth media, a 33-fold increase in lysine acetylation was previously determined compared with growth in media without glucose. Using our SWATH stoichiometry quantification method, we obtained a similar result, revealing 9.2% acetyl occupancy in bacteria grown with glucose versus 0.4% occupancy when those same bacteria were grown without glucose (~23-fold increase, Supplementary Table S5).

For additional confirmation as well as demonstrate the use of targeted MS approaches to make the same measurements, we determined occupancy levels using PRM), as described in a recent paper [46]. Using PRM acquisition, all MS2 fragment ions, including common fragment ions of the same mass, can be used for stoichiometry calculations, as their separate precursor ions are isolated at unit resolution (Supplementary Table S5). Fragment ion peak areas from PRM experiments of the peptide containing GPMA K-100 reveal low acetyl occupancy of ~0.2% when bacteria were grown without glucose (Supplementary Figure S3d). However, when *E. coli* growth media was supplemented with glucose, occupancies of 9.6% were determined for this site (Supplementary Figure S3c), which is essentially the same value obtained by SWATH (Supplementary Table S5). This measured change in acetyl stoichiometry represents a 48-fold increase in acetylation at this GPMA site K-100 upon growth with added glucose, which is similar to the 33-fold increase we previously determined using label-free quantification from the lysine acetylation enrichment workflow. After the initial untargeted SWATH analysis, such targeted PRM experiments could be implemented as quantitative, high-throughput assays to process many different samples and conditions.

A relatively high occupancy of 9% for GPMA at K-100 is of special functional interest as this lysine is located in the binding site for the enzyme’s substrate, 2-phospho-D-glycerate (Supplementary Figure S3b). Three independent biological replicates of *E. coli* grown with high glucose were prepared and processed, and Supplementary Figure S4a shows a selected subset of sites for which acetylation occupancy was calculated for acetylation sites from proteins ClpB, DnaK, and LRP using this new SWATH MS2 quantification method (a more complete table of sites is provided as Supplementary Table S4). Reproducibility between biological replicates was good for both low and high stoichiometries, indicating a robust workflow. For example, lysine acetylation occupancy at K-25 of LRP protein in three biological replicates was measured as 5.5%, 4.6%, and 4.6%.

**Figure 4:**
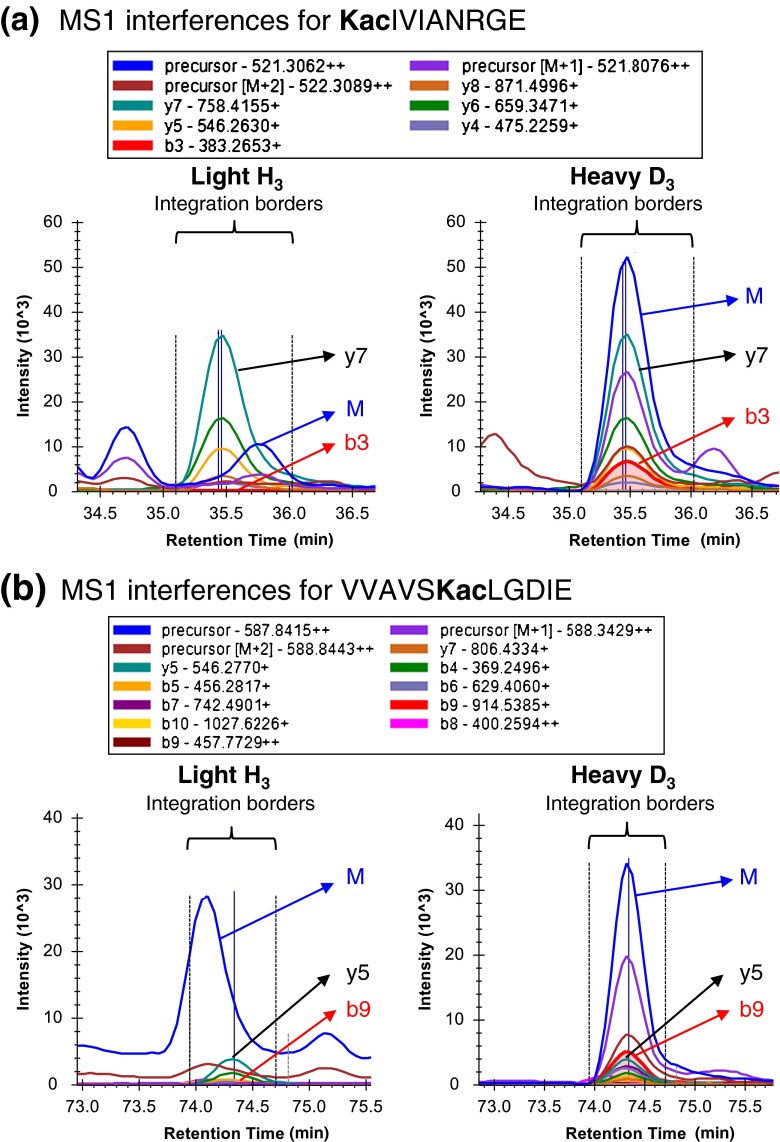
Demonstration of MS1 interferences. **(a)** Precursor and fragment ion chromatograms were extracted from light/heavy precursor ions pairs at *m/z* 521.30 and *m/z* 522.80 for the acetylated peptide **Kac**IVIANRGE containing K-4 in ACCC protein. Strong interferences in the light MS1 signal that did not co-elute with the extracted fragment ions led to an overestimated MS1-based lysine acetylation occupancy 14.3%. The fragment ion XICs, specifically from the differentiating b_3_ ion, indicated an acetylation occupancy of 0.6%. **(b)** Precursor and fragment ion chromatograms were extracted from light/heavy precursor ions pairs at *m/z* 586.33 and *m/z* 587.84 for the acetylated peptide VVAVS**Kac**LGDIE containing K-48 in GRCA protein. Strong interferences in the light MS1 signal that clearly did not co-elute with other ions led to an overestimated MS1-based lysine acetylation occupancy of 39.1%. The fragment ion XICs, specifically from the differentiating b_9_ ion, indicated an acetylation occupancy of 3.2%

As described for BSA validation experiments, another benefit from using SWATH analysis is that we can directly compare occupancy calculations using MS1 scans acquired as part of each SWATH cycle with the MS2-based fragment ion calculations. In some cases, MS1- and MS2-based calculations yielded very similar results for many sites; however, the MS1-based results showed higher, and likely inflated, estimates of occupancy compared with those based on MS2 ion intensities (Supplementary Table S4). MS1-based occupancy calculations also showed more variation among biological replicates, especially when they differed from the SWATH MS2-based fragment ion results (Supplementary Figure S4b). Lack of precision and accuracy observed from MS1-based calculations is likely caused by incorrect MS1 measurements due to random interferences and/or noise. While MS1 measurements can yield acceptable results for strong signals with little or no interferences, in this study, the moderate complexity of the *E. coli* proteome made the MS1 signals prone to interferences. Stoichiometry determinations based on MS2 signals are less prone to interferences, as the MS2 space is less crowded, particularly when using variable window SWATH acquisitions. In addition, because multiple fragment ions are measured for each peptide, interferences can more easily be detected as outliers in disagreement with other fragments, allowing for selection of only the most robust fragment ions for quantification. Figure 4 shows some obvious MS1 interferences in the light, endogenous channel for acetylated or succinylated peptides, whereas MS2 fragment ions allowed interference-free quantification. Overall, MS2-based fragment ion measurements for occupancy appear more reliable and show relatively low stoichiometry measurements for both acetylated and succinylated peptides (Supplementary Tables S4 and S6).

In parallel to the acetylation occupancy experiments in *E. coli*, experiments using our new succinylation workflow were applied to wild type *E. coli* bacteria grown under conditions similar to those use for the acetylation studies. Similar to lysine acetylation, the majority of lysine succinylation modifications in wild type *E.coli* proteins showed less than 2% occupancy (Supplementary Table S6). That said, we anticipate that there are conditions where lysine succinylation occupancies will be elevated as observed for glucose-induced acetylation occupancy. For example, it is expected that any condition that elevates succinyl-CoA levels should yield increased succinyllysine stoichiometries. A similar scenario is possible in mutants deleted for the gene that encodes the Sir2 homolog, CobB, which has been shown to exhibit both deacetylase and desuccinylase activities [17, 21, 34].

In an effort to improve accuracy of MS1 measurements, some of the previously reported stoichiometry determinations attempted to reduce complexity of the samples by offline fractionation. Here, we used our *E. coli* succinylation site occupancy experiments to illustrate interference reduction afforded by both the use of SWATH-generated fragment ions for quantification and peptide pre-fractionation by offline bRP (Supplementary Figure S6). Some examples for succinylation occupancy that clearly showed the reduction of interferences upon fractionation are displayed in Supplementary Table S6 and Supplementary Figure S5. The mean succinyl occupancy measured only from precursor signal in unfractionated sample was 5.4% ± 9.6% (Supplementary Figure S6). Using fragment ion signal from the same acquisitions to calculate occupancy, however, resulted in an average of 3.0% ± 5.5%, which clearly demonstrates how possible MS1 interferences can inflate reported occupancy. Furthermore, the reduction of interferences due to fractionation is evident both in the MS1- and MS2-measured succinyl occupancies; the average occupancy measured by MS1 in the bRP fractionated samples was 1.5% ± 4.9%, and the average occupancy measured by MS2 in the bRP fractionated samples was 0.7% ± 0.9% (Supplementary Figure S6). The improvements in quantification from fractionated samples appear to result from both the reduction of sample complexity and the ability to load more sample; thus, peptide fractionation increases signal and reduces complexity.

**Figure 5:**
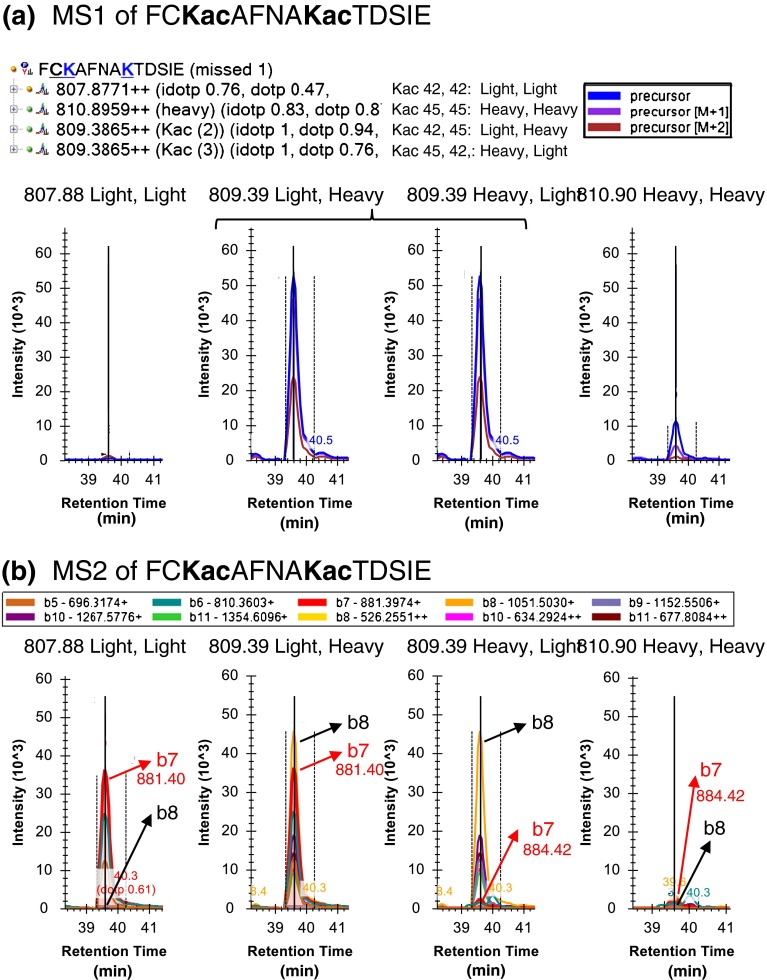
Stoichiometry calculation for peptides containing two Lysine residues. **(a)** Skyline target tree can be populated with all possible light (L) and heavy (H) acetyl permutations for peptide FC**Kac**AFNA**Kac**TDSIE from ribosomal RL11 protein at *m/z* 807.88 (LL), *m/z* 810.90 (HH), and the mixed species at *m/z* 809.29 (LH and HL). Precursor ion XICs are shown for all four species, although the mixed species LH and HL cannot be differentiated using precursor m/z alone. **(b)** Fragment ion XICs can differentiate the occupancy for both lysine acetylation sites. Fragment ion b_7_, which contains only the first lysine residue, K-40, was used to determine a stoichiometry of 92.3%. The b_8_ ion, containing both lysines, was used to calculate a low stoichiometry of 1.6% for the second lysine site, K-45, present in this peptide

Peptides containing multiple lysines present an additional challenge when determining acylation occupancy, which our fragment ion-based quantification strategy is uniquely well-suited to determine. An example of stoichiometry determination for a peptide containing two acetylated lysine residues from ribosomal protein RL11 is shown in Figure 5. Skyline allows for synchronized quantification of any combination of light and heavy acetylated lysine residues, i.e., an unlabeled peptide (light/light), singly-labeled mixed species (light/heavy and heavy/light), and the doubly-labeled version (heavy/heavy). Although the precursor ion signal does reflect the total level endogenous acetylation for the singly-labeled mixed peptide (Figure 5a), endogenous acylation at the first lysine cannot be differentiated from the endogenous acylation at the second lysine. However, using differentiating fragment ions it is possible to determine endogenous acetylation levels for each lysine site independently (Figure 5b). In this specific example, the *b*_*7*_ ion, which contains only the first acetylation site, has strong light (endogenous) signal (*m/z* 881.4), whereas the corresponding heavy transition (*m/z* 884.4) is much weaker. These results indicated that the high level of endogenous acetylation observed was on the first lysine (K-40) with a high stoichiometry of 92.3%. Supplementary Figure S7a demonstrates how to set up the Skyline document for peptides containing two Kac residues. In addition, we have developed an in-house algorithm, the StoichiolyzeR, to automate the determination of acylation site stoichiometries from peptides with multiple lysines. Supplementary Figure S7b indicates specific details and considerations regarding stoichiometry calculations for ‘double Kac’ peptides.

## Conclusions

In this study, we present a novel method to quantify site-specific acetylation and succinylation occupancy that can be applied to an entire proteome. Similar to previous methods, our strategy is based on chemical acylation of all unmodified lysines, but in contrast our approach has several advantages due to the use of more comprehensive SWATH acquisition. We demonstrate how signal interferences, particularly in MS1 scans, can lead to overestimation of site occupancies. More importantly, we show that interferences can be decreased by using a SWATH-MS2-based quantification strategy. Even when interferences were present in some MS2 scans, access to comprehensive MS2 data from SWATH acquisitions allows selection of a different fragment ion from the same peptide that would also differentiate the light and heavy peptide forms, and stoichiometries can still be determined. However, precursor ion interferences can rarely be corrected in post-acquisition analysis.

Precursor interferences may become less of an issue for next generation high-resolution instruments generating exceedingly high MS1 resolution; however, there are additional advantages unique to fragment ion quantification from SWATH acquisitions. First, when using precursor ion quantification only, it is not possible to determine stoichiometry for peptides containing multiple lysine residues; using fragment ion quantification from SWATH data, we can measure stoichiometry for peptides containing up to three different lysines with our novel algorithm StoichiolyzeR. Second, SWATH data containing full scan MS and MS/MS information significantly improves confidence in peak assignment during data processing. Third, SWATH data collection and analysis using the combination of Skyline and our StoichiolyzeR package can be applied to any similar stable isotope-based workflow that traditionally uses precursor signal quantification to improve quantification accuracy, such as SILAC or dimethyl labeling. Finally, this type of fragment-specific quantification can be applied to new areas such as PTM localization.

In addition to the advantages of using SWATH as discussed above, we found that fractionation of peptides prior to mass spectrometric analysis greatly improves our measurements, especially for complex samples. Most of the lysine-acetylation and lysine-succinylation stoichiometries measured from *E. coli* proteins using our SWATH-MS2 methodology were relatively low (<5%). We determined acetylation occupancies between 0% and 2% for 84% of the monitored sites, occupancies between 2% and 5% for 12% of the sites, occupancies between 5% and 10% for 3% of the sites, and we observed only 1% of sites with occupancies >10% (Supplementary Figure S8), for our given experimental conditions. Stoichiometry assessments may guide researchers towards these acylation hotspots and possibly provide valuable information for which acylation sites to invest in biological follow-up experiments, for example, site-directed mutagenesis of the acylation site. In addition, we outlined interesting mass spectrometric follow-up workflows that can be performed, such as PRM-MS. For example, after an initial global SWATH analysis, highly multiplexed, targeted PRM-MS acquisitions [46] of most interesting targets (i.e., high site occupancy) could be implemented as high throughput, quantitative assay to process many different samples and conditions.

We believe it is important to determine occupancy for multiple forms of acylation, as it has been shown that many succinylation sites overlap extensively with acetylation [11, 55]. An assumption of previous acetylation stoichiometry studies was that each measured peptide is only modified by acetylation, which we know from several studies does not have to be a true. Even in this study, we are likely overestimating site occupancy (slightly) because of other unaccounted lysine modifications at the same site, such as ubiquitination and methylation. However, by extending the method to also measure succinylation stoichiometry, we raise the possibility of using several per-acylation methods in parallel to get more accurate readings of actual endogenous site occupancy among several modifications.

Several aspects of our unique data analysis pipeline facilitate this new workflow. The use of Skyline’s graphical user interface for stoichiometry calculation provides an easy way to assess and confirm quality of measurements. Light and heavy signals are always integrated together over the same elution time window (‘integrate all’ feature in Skyline). In addition, using the DIA-Umpire signal extraction algorithm to generate pseudo MS/MS spectra allowed us to import SWATH acquisition-derived identifications from database searches into Skyline, which then displays a line in the chromatograms marking the time of identification, referred to as “ID line.” This ensures that the correct peak for occupancy calculation is selected in all cases because the heavy, non-endogenous signal is almost always sufficiently abundant to be identified by the database search. Finally, the StoichiolyzeR package provides an easy way to calculate the stoichiometry, including from peptides containing multiple lysine residues.

Overall, we present methods for the determination of acetylation and succinylation stoichiometry that are robust and less prone to ion interferences than MS1-only measurements. Data obtained from these protocols should provide more accurate insights into dynamic acylation effects on proteins under different biological conditions. Even though we find that in our system most sites have low acylation stoichiometries, these modifications may still play a very significant functional role in modulating pathways and networks. For example, low-level lysine acylation could exert significant effects on the formation and/or stability of protein–protein interactions and protein complexes, especially for metabolic proteins that often form homo-multimers with cooperative activity. Alternately, these low-level modifications could have indirect functional effects, acting as a sink for acyl-CoA during nutrient excess, and by regulating the pool of NAD+, which is degraded by sirtuin-coupled deacylation.

## Electronic supplementary material

Below is the link to the electronic supplementary material.

ESM 1 (PDF 6.37 mb)

Supplementary Figure S1 (XLSX 518 kb)

Supplementary Figure S2 (XLSX 12 kb)

Supplementary Figure S3 (XLSX 113 kb)

Supplementary Figure S4 (XLSX 42 kb)

Supplementary Figure S5 (XLSX 15 kb)

Supplementary Figure S6 (XLSX 24 kb)

## References

[1] Glozak MA, Seto E (2007). Histone deacetylases and cancer. Oncogene.

[2] Yang XJ, Seto E (2008). Lysine acetylation: codified crosstalk with other posttranslational modifications. Mol. Cell.

[3] Choudhary C, Weinert BT, Nishida Y, Verdin E, Mann M (2014). The growing landscape of lysine acetylation links metabolism and cell signalling. Nat. Rev. Mol. Cell Biol..

[4] Bannister AJ, Kouzarides T (2011). Regulation of chromatin by histone modifications. Cell Res..

[5] Verdin E, Ott M (2015). Fifty years of protein acetylation: from gene regulation to epigenetics, metabolism, and beyond. Nat. Rev. Mol. Cell Biol..

[6] Choudhary C, Kumar C, Gnad F, Nielsen ML, Rehman M, Walther TC, Olsen JV, Mann M (2009). Lysine acetylation targets protein complexes and co-regulates major cellular functions. Science.

[7] Kim SC, Sprung R, Chen Y, Xu Y, Ball H, Pei J, Cheng T, Kho Y, Xiao H, Xiao L, Grishin NV, White M, Yang XJ, Zhao Y (2006). Substrate and functional diversity of lysine acetylation revealed by a proteomics survey. Mol. Cell.

[8] Rardin MJ, Newman JC, Held JM, Cusack MP, Sorensen DJ, Li B, Schilling B, Mooney SD, Kahn CR, Verdin E, Gibson BW (2013). Label-free quantitative proteomics of the lysine acetylome in mitochondria identifies substrates of SIRT3 in metabolic pathways. Proc. Natl. Acad. Sci. U. S. A..

[9] Still AJ, Floyd BJ, Hebert AS, Bingman CA, Carson JJ, Gunderson DR, Dolan BK, Grimsrud PA, Dittenhafer-Reed KE, Stapleton DS, Keller MP, Westphall MS, Denu JM, Attie AD, Coon JJ, Pagliarini DJ (2013). Quantification of mitochondrial acetylation dynamics highlights prominent sites of metabolic regulation. J. Biol. Chem..

[10] Nishida Y, Rardin MJ, Carrico C, He W, Sahu AK, Gut P, Najjar R, Fitch M, Hellerstein M, Gibson BW, Verdin E (2015). SIRT5 regulates both cytosolic and mitochondrial protein malonylation with glycolysis as a major target. Mol. Cell.

[11] Rardin MJ, He W, Nishida Y, Newman JC, Carrico C, Danielson SR, Guo A, Gut P, Sahu AK, Li B, Uppala R, Fitch M, Riiff T, Zhu L, Zhou J, Mulhern D, Stevens RD, Ilkayeva OR, Newgard CB, Jacobson MP, Hellerstein M, Goetzman ES, Gibson BW, Verdin E (2013). SIRT5 regulates the mitochondrial lysine succinylome and metabolic networks. Cell Metab..

[12] Park J, Chen Y, Tishkoff DX, Peng C, Tan M, Dai L, Xie Z, Zhang Y, Zwaans BM, Skinner ME, Lombard DB, Zhao Y (2013). SIRT5-mediated lysine desuccinylation impacts diverse metabolic pathways. Mol. Cell.

[13] Peng C, Lu Z, Xie Z, Cheng Z, Chen Y, Tan M, Luo H, Zhang Y, He W, Yang K, Zwaans BM, Tishkoff D, Ho L, Lombard D, He TC, Dai J, Verdin E, Ye Y, Zhao Y (2011). The first identification of lysine malonylation substrates and its regulatory enzyme. Mol. Cell. Proteomics.

[14] Hirschey MD, Zhao Y (2015). Metabolic regulation by lysine malonylation, succinylation, and glutarylation. Mol. Cell. Proteomics.

[15] Baeza J, Dowell JA, Smallegan MJ, Fan J, Amador-Noguez D, Khan Z, Denu JM (2014). Stoichiometry of site-specific lysine acetylation in an entire proteome. J. Biol. Chem..

[16] Castano-Cerezo S, Bernal V, Post H, Fuhrer T, Cappadona S, Sanchez-Diaz NC, Sauer U, Heck AJ, Altelaar AF, Canovas M (2014). Protein acetylation affects acetate metabolism, motility and acid stress response in Escherichia coli. Mol. Syst. Biol..

[17] Castano-Cerezo S, Bernal V, Rohrig T, Termeer S, Canovas M (2015). Regulation of acetate metabolism in *Escherichia coli* BL21 by protein N(epsilon)-lysine acetylation. Appl. Microbiol. Biotechnol..

[18] Hu LI, Chi BK, Kuhn ML, Filippova EV, Walker-Peddakotla AJ, Basell K, Becher D, Anderson WF, Antelmann H, Wolfe AJ (2013). Acetylation of the response regulator RcsB controls transcription from a small RNA promoter. J. Bacteriol..

[19] Lima BP, Antelmann H, Gronau K, Chi BK, Becher D, Brinsmade SR, Wolfe AJ (2011). Involvement of protein acetylation in glucose-induced transcription of a stress-responsive promoter. Mol. Microbiol..

[20] Lima BP, Thanh Huyen TT, Basell K, Becher D, Antelmann H, Wolfe AJ (2012). Inhibition of acetyl phosphate-dependent transcription by an acetylatable lysine on RNA polymerase. J. Biol. Chem..

[21] Colak G, Xie Z, Zhu AY, Dai L, Lu Z, Zhang Y, Wan X, Chen Y, Cha YH, Lin H, Zhao Y, Tan M (2013). Identification of lysine succinylation substrates and the succinylation regulatory enzyme CobB in Escherichia coli. Mol. Cell. Proteomics.

[22] Kuhn ML, Zemaitaitis B, Hu LI, Sahu A, Sorensen D, Minasov G, Lima BP, Scholle M, Mrksich M, Anderson WF, Gibson BW, Schilling B, Wolfe AJ (2014). Structural, kinetic, and proteomic characterization of acetyl phosphate-dependent bacterial protein acetylation. PLoS One.

[23] Schilling B, Christensen D, Davis R, Sahu AK, Hu LI, Walker-Peddakotla A, Sorensen DJ, Zemaitaitis B, Gibson BW, Wolfe AJ (2015). Protein acetylation dynamics in response to carbon overflow in Escherichia coli. Mol. Microbiol..

[24] Weinert BT, Iesmantavicius V, Wagner SA, Scholz C, Gummesson B, Beli P, Nystrom T, Choudhary C (2013). Acetyl-phosphate is a critical determinant of lysine acetylation in E. coli. Mol. Cell.

[25] Wagner GR, Payne RM (2013). Widespread and enzyme-independent Nepsilon-acetylation and Nepsilon-succinylation of proteins in the chemical conditions of the mitochondrial matrix. J. Biol. Chem..

[26] Weinert BT, Iesmantavicius V, Moustafa T, Scholz C, Wagner SA, Magnes C, Zechner R, Choudhary C (2014). Acetylation dynamics and stoichiometry in *Saccharomyces cerevisiae*. Mol. Syst. Biol..

[27] Michishita E, Park JY, Burneskis JM, Barrett JC, Horikawa I (2005). Evolutionarily conserved and nonconserved cellular localizations and functions of human SIRT proteins. Mol. Biol. Cell.

[28] Schwer B, North BJ, Frye RA, Ott M, Verdin E (2002). The human silent information regulator (Sir)2 homologue hSIRT3 is a mitochondrial nicotinamide adenine dinucleotide-dependent deacetylase. J. Cell Biol..

[29] He W, Newman JC, Wang MZ, Ho L, Verdin E (2012). Mitochondrial sirtuins: regulators of protein acylation and metabolism. Trends Endocrinol. Metab..

[30] Hirschey MD, Shimazu T, Goetzman E, Jing E, Schwer B, Lombard DB, Grueter CA, Harris C, Biddinger S, Ilkayeva OR, Stevens RD, Li Y, Saha AK, Ruderman NB, Bain JR, Newgard CB, Farese RV, Alt FW, Kahn CR, Verdin E (2010). SIRT3 regulates mitochondrial fatty-acid oxidation by reversible enzyme deacetylation. Nature.

[31] Schwer B, Eckersdorff M, Li Y, Silva JC, Fermin D, Kurtev MV, Giallourakis C, Comb MJ, Alt FW, Lombard DB (2009). Calorie restriction alters mitochondrial protein acetylation. Aging Cell.

[32] Zhang J, Sprung R, Pei J, Tan X, Kim S, Zhu H, Liu CF, Grishin NV, Zhao Y (2009). Lysine acetylation is a highly abundant and evolutionarily conserved modification in Escherichia coli. Mol. Cell. Proteomics.

[33] Yu BJ, Kim JA, Moon JH, Ryu SE, Pan JG (2008). The diversity of lysine-acetylated proteins in *Escherichia coli*. J. Microbiol. Biotechnol..

[34] AbouElfetouh A, Kuhn ML, Hu LI, Scholle MD, Sorensen DJ, Sahu AK, Becher D, Antelmann H, Mrksich M, Anderson WF, Gibson BW, Schilling B, Wolfe AJ (2015). The *E. coli* sirtuin CobB shows no preference for enzymatic and nonenzymatic lysine acetylation substrate sites. MicrobiologyOpen.

[35] Svinkina T, Gu H, Silva JC, Mertins P, Qiao J, Fereshetian S, Jaffe JD, Kuhn E, Udeshi ND, Carr SA (2015). Deep, quantitative coverage of the lysine acetylome using novel anti-acetyl-lysine antibodies and an optimized proteomic workflow. Mol. Cell. Proteomics.

[36] Olsen JV, Vermeulen M, Santamaria A, Kumar C, Miller ML, Jensen LJ, Gnad F, Cox J, Jensen TS, Nigg EA, Brunak S, Mann M (2010). Quantitative phosphoproteomics reveals widespread full phosphorylation site occupancy during mitosis. Sci. Signal..

[37] Colak G, Pougovkina O, Dai L, Tan M, Te Brinke H, Huang H, Cheng Z, Park J, Wan X, Liu X, Yue WW, Wanders RJ, Locasale JW, Lombard DB, de Boer VC, Zhao Y (2015). Proteomic and biochemical studies of lysine malonylation suggest its malonic aciduria-associated regulatory role in mitochondrial function and fatty acid oxidation. Mol. Cell. Proteomics.

[38] Weinert BT, Moustafa T, Iesmantavicius V, Zechner R, Choudhary C (2015). Analysis of acetylation stoichiometry suggests that SIRT3 repairs nonenzymatic acetylation lesions. EMBO J..

[39] Nakayasu, E.S., Wu, S., Sydor, M.A., Shukla, A.K., Weitz, K.K., Moore, R.J., Hixson, K.K., Kim, J.S., Petyuk, V.A., Monroe, M.E., Pasa-Tolic, L., Qian, W.J., Smith, R.D., Adkins, J.N., Ansong, C.: A method to determine lysine acetylation stoichiometries. Int. J. Proteomics **2014**(730725) (2014) 10.1155/2014/730725 PMC4131070 25143833

[40] Zhou T, Chung YH, Chen J, Chen Y (2016). Site-specific identification of lysine acetylation stoichiometries in mammalian cells. J. Proteome Res..

[41] Zhang R, Sioma CS, Thompson RA, Xiong L, Regnier FE (2002). Controlling deuterium isotope effects in comparative proteomics. Anal. Chem..

[42] Rardin MJ, Schilling B, Cheng LY, MacLean BX, Sorensen DJ, Sahu AK, MacCoss MJ, Vitek O, Gibson BW (2015). MS1 peptide ion intensity chromatograms in MS2 (SWATH) data-independent acquisitions. Improving post-acquisition analysis of proteomic experiments. Mol. Cell. Proteomics.

[43] Keshishian H, Addona T, Burgess M, Kuhn E, Carr SA (2007). Quantitative, multiplexed assays for low abundance proteins in plasma by targeted mass spectrometry and stable isotope dilution. Mol. Cell. Proteomics.

[44] Wang Y, Yang F, Gritsenko MA, Wang Y, Clauss T, Liu T, Shen Y, Monroe ME, Lopez-Ferrer D, Reno T, Moore RJ, Klemke RL, Camp DG, Smith RD (2011). Reversed-phase chromatography with multiple fraction concatenation strategy for proteome profiling of human MCF10A cells. Proteomics.

[45] Schilling B, Rardin MJ, MacLean BX, Zawadzka AM, Frewen BE, Cusack MP, Sorensen DJ, Bereman MS, Jing E, Wu CC, Verdin E, Kahn CR, MacCoss MJ, Gibson BW (2012). Platform-independent and label-free quantitation of proteomic data using MS1 extracted ion chromatograms in skyline: application to protein acetylation and phosphorylation. Mol. Cell. Proteomics.

[46] Schilling B, MacLean B, Held JM, Sahu AK, Rardin MJ, Sorensen DJ, Peters T, Wolfe AJ, Hunter CL, MacCoss MJ, Gibson BW (2015). Multiplexed, scheduled, high-resolution parallel reaction monitoring on a full scan QqTOF instrument with integrated data-dependent and targeted mass spectrometric workflows. Anal. Chem..

[47] Tsou CC, Avtonomov D, Larsen B, Tucholska M, Choi H, Gingras AC, Nesvizhskii AI (2015). DIA-Umpire: comprehensive computational framework for data-independent acquisition proteomics. Nat. Methods.

[48] Perkins DN, Pappin DJ, Creasy DM, Cottrell JS (1999). Probability-based protein identification by searching sequence databases using mass spectrometry data. Electrophoresis.

[49] Kim S, Pevzner PA (2014). MS-GF+ makes progress towards a universal database search tool for proteomics. Nat. Commun..

[50] Keller A, Nesvizhskii AI, Kolker E, Aebersold R (2002). Empirical statistical model to estimate the accuracy of peptide identifications made by MS/MS and database search. Anal. Chem..

[51] Shteynberg D, Deutsch EW, Lam H, Eng JK, Sun Z, Tasman N, Mendoza L, Moritz RL, Aebersold R, Nesvizhskii AI (2011). iProphet: multi-level integrative analysis of shotgun proteomic data improves peptide and protein identification rates and error estimates. Mol. Cell. Proteomics.

[52] MacLean B, Tomazela DM, Shulman N, Chambers M, Finney GL, Frewen B, Kern R, Tabb DL, Liebler DC, MacCoss MJ (2010). Skyline: an open source document editor for creating and analyzing targeted proteomics experiments. Bioinformatics.

[53] Chambers MC, MacLean B, Burke R, Amodei D, Ruderman DL, Neumann S, Gatto L, Fischer B, Pratt B, Egertson J, Hoff K, Kessner D, Tasman N, Shulman N, Frewen B, Baker TA, Brusniak MY, Paulse C, Creasy D, Flashner L, Kani K, Moulding C, Seymour SL, Nuwaysir LM, Lefebvre B, Kuhlmann F, Roark J, Rainer P, Detlev S, Hemenway T, Huhmer A, Langridge J, Connolly B, Chadick T, Holly K, Eckels J, Deutsch EW, Moritz RL, Katz JE, Agus DB, MacCoss M, Tabb DL, Mallick P (2012). A cross-platform toolkit for mass spectrometry and proteomics. Nat. Biotechnol..

[54] Riordan JF, Vallee BL (1972). 41 Acetylation. Methods Enzymol..

[55] Weinert BT, Scholz C, Wagner SA, Iesmantavicius V, Su D, Daniel JA, Choudhary C (2013). Lysine succinylation is a frequently occurring modification in prokaryotes and eukaryotes and extensively overlaps with acetylation. Cell Rep..

