# Supplementary material for: Quantification of Lysine Acetylation and Succinylation Stoichiometry in Proteins Using Mass Spectrometric Data-Independent Acquisitions (SWATH)
Source: J Am Soc Mass Spectrom. 2016 Sep 2;27(11):1758–71. doi: 10.1007/s13361-016-1476-z (PMC5059418; doi:10.1007/s13361-016-1476-z)

Supplementary Figures

Quantification of Lysine Acetylation and Succinylation Stoichiometry in Proteins Using Mass Spectrometric Data-Independent Acquisitions (SWATH)

Running Title: Lysine Acylation Site Occupancy

Jesse G. Meyer,[1] Alexandria K. D'Souza,[1] Dylan J. Sorensen,[1] Matthew J. Rardin,[2] Alan J. Wolfe,[3] Bradford W. Gibson,[1,4] Birgit Schilling[1]

[1]Buck Institute for Research on Aging, Novato, CA 94945, USA

[2]Amgen, South San Francisco, CA 94080, USA

[3]Department of Microbiology and Immunology, Stritch School of Medicine, Health Sciences Division, Loyola University Chicago, Maywood, IL 60153, USA

[4]Department of Pharmaceutical Chemistry, University of California, San Francisco, CA 94143, USA

Correspondence to: Bradford W. Gibson; email: bgibson@buckinstitute.org, and Birgit Schilling; email: bschilling@buckinstitute.org

Electronic supplementary material provided

Table of Contents

- **Supplementary Figure S1.** Reaction efficiency and byproducts of chemical peracetylation reaction.
- **Supplementary Figure S2.** Skyline target tree and BSA acetylation stoichiometry.
- **Supplementary Figure S3.** Additional lysine acetylation site occupancy examples from *E. coli* whole cell lysate.
- **Supplementary Figure S4.** Stoichiometry examples.
- **Supplementary Figure S5.** Assessing potential (MS1) interferences during stoichiometry analysis and reducing complexity of samples by offline fractionation.
- **Supplementary Figure S6.** The role of sample fractionation for stoichiometry analysis.
- **Supplementary Figure S7.** Example set-up of peptides containing two acyl groups in Skyline's modification interface.
- **Supplementary Figure S8.** Distribution of acylation stoichiometry measurements as determined from *E. coli* samples.

Supplementary Figure S1.

Reaction efficiency and byproducts of chemical peracetylation reaction. Peak area for one peptide from BSA in unmodified, lysine acetylated, and both lysine and serine acetylated forms as a percentage of either the maximum unmodified area (green bars) or maximum acetyl-lysine area (blue and red bars). **(a)** Percent peak areas with y-axis scaled from 0-100% showing that the unmodified peptide is chemically reacted immediately to form the acetylated species. **(b)** Zoom in: percent peak area with y-axis scaled from 0-1% to show very low abundance species (<0.2% in all cases for). Although the O-acetylated species is identified as minor signal, the extracted peak area from this side product (serine acetylation) is less than 0.25% of the maximum peak area from the lysine-acetylated peptide.

Supplemental Figure 1

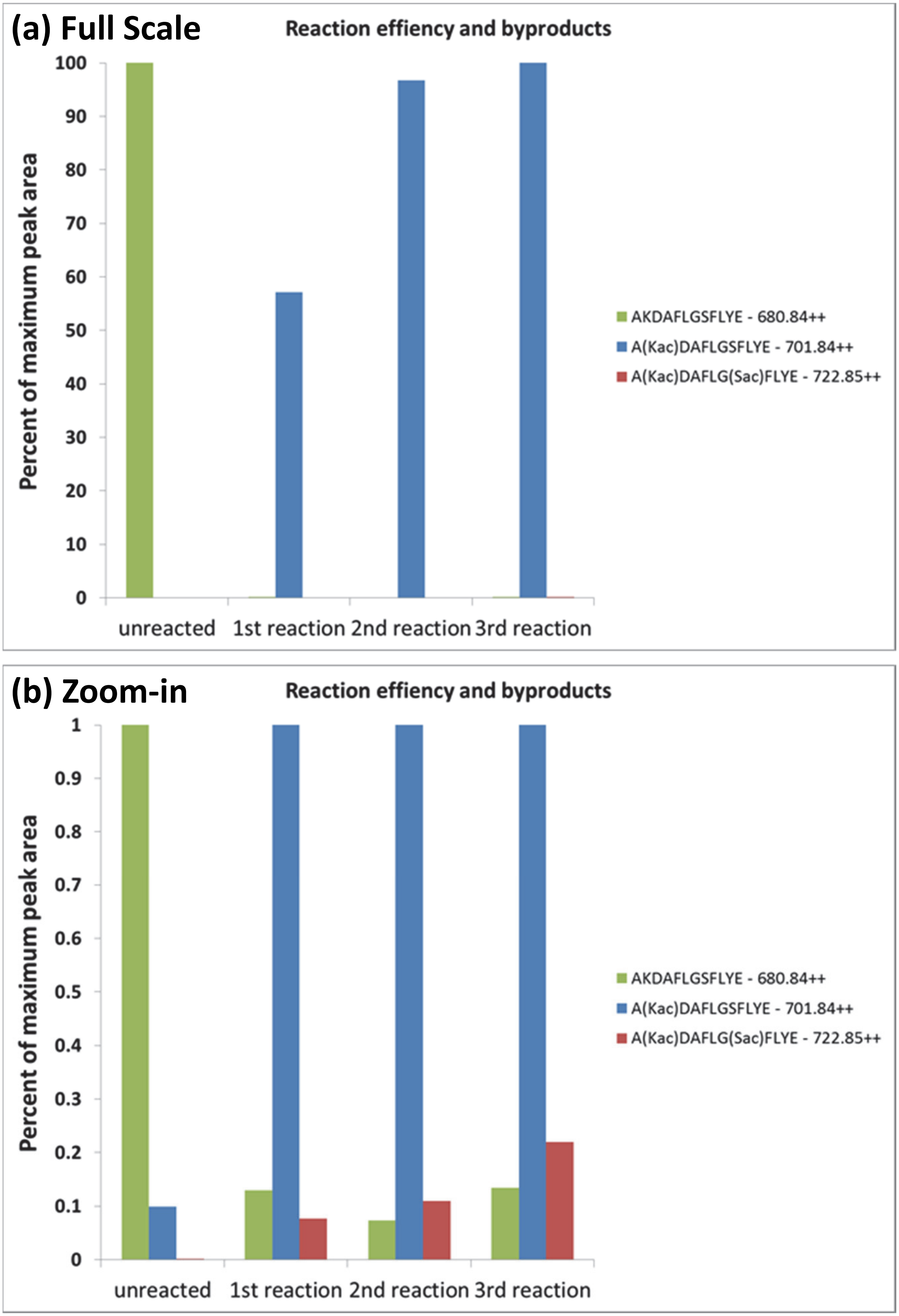

Supplementary Figure S2.

(a) An example of the Skyline target tree is displayed for the light and heavy peptide pair corresponding to peptide TYVP**Kac**AFDE from BSA at *m/z* 556.27 and 557.78. Differentiating light and heavy ions are boxed in red and blue rectangles, respectively. For the light peptide and corresponding transitions, L/H ratios are indicated in parenthesis that serve as an initial estimation for occupancy (later, 'L/(L+H)' ratios are calculated outside the Skyline environment). Individual differentiating ions that contain the acetyl group yield similar occupancy estimations; fragment ions that do not contain the acetyl group show an L/H ratio of '1'. **(b)** Plot showing how the observed rank of precursor and fragment ions influences the resulting calculated stoichiometry L/(L+H) from acetylated BSA peptides identified in our experiments that i) showed reasonable signal to noise ratios and ii) contained only one lysine residue. Ranking is determined for precursor ions based on the theoretical natural isotopic distribution, while the ranking for fragment ions was based on observed MS/MS spectra from spectral libraries. The highest ranked precursor ions yield most reasonable occupancy estimations for this endogenous BSA sample with near the expected ~0% endogenous acetylation levels. Similarly, MS2-based stoichiometry calculations using rank 1 and rank 2 fragment ions show lowest, more accurate, occupancy measurements. Lower ranked fragment ions as well as lower ranked precursor ions (with lower relative intensity) appear to be less accurate for the occupancy estimations likely due to worse interferences and ion statistics.

Supplemental Figure 2

(a) Skyline target tree light and heavy acetylated BSA peptide

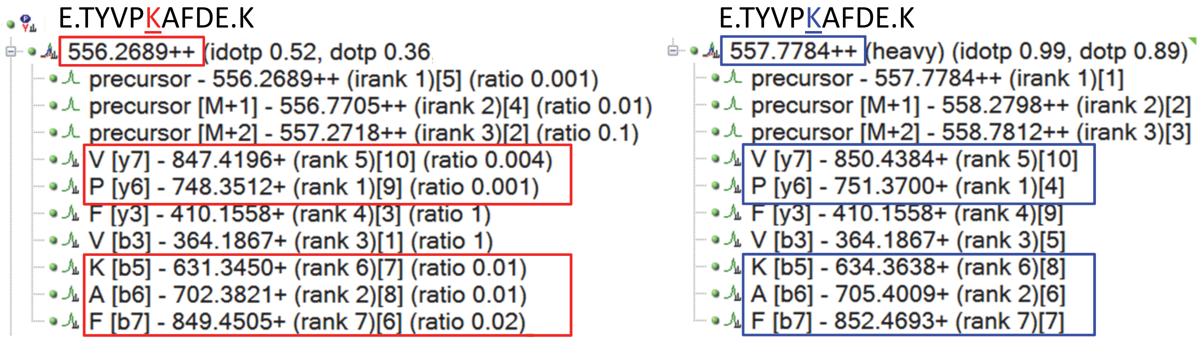

(b) Stoichiometry calculations for non-acetylated BSA

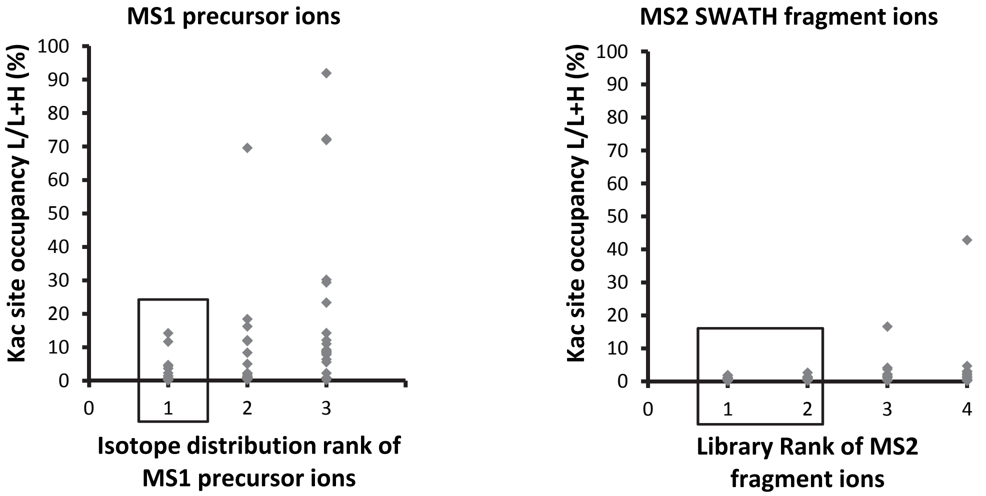

Supplementary Figure S3.

Additional lysine acetylation site occupancy examples from *E. coli* whole cell lysate. **(a)** A relatively high stoichiometry was determined for acetylated peptide RHYGALQGLN**Kac**AE corresponding to K-100 in protein GPMA. XICs are shown for fragment ions resulting from light and heavy precursor ions at *m/z* 749.89 and *m/z* 751.40, respectively. The highest ranked differentiating ion, b_{11}, is used for occupancy calculations. The displayed Skyline trees indicate L/H ratios determined for each of the fragment ions as displayed for the light peptide. Skyline reports an L/H ratio of 0.07 (7%) for the differentiating ion $b_{11,}$ which provides a quick assessment when reviewing the Skyline document. For fragment ions do not contain the acetyl modification, Skyline reports an L/H ratio of 1, as expected. **(b)** The three-dimensional structure for GPMA was visualized with Pymol, and lysine acetylation sites are marked with color. K-100 is indicated, which showed a relatively high stoichiometry when growth media was supplemented with glucose. **(c)** Skyline tree screenshots for the same acetylated peptide monitored either by parallel reaction monitoring (PRM) or SWATH acquisitions obtained from *E. coli* bacteria grown under glucose supplement conditions. While for SWATH acquisitions only differentiating ions can be used with different *m/z* fragment ion values (because precursor ions for the light and heavy pairs were co-fragmented in the same SWATH window), all fragment ions measured from PRM acquisitions can be used for occupancy calculations. PRM fragment ions indicate L/H ratio of ~9% **(d)** Screenshots of the Skyline tree for the same peptide monitored by either PRM or SWATH, except measured from *E. coli* bacteria grown <u>without</u> glucose supplementation. PRM fragment ions indicate L/H ratio of ~0.02%.

Supplemental Figure 3

(a) GPMA protein, Kac – 100, high stoichiometry (~ 7%)

GPMA: 2,3-bisphosphoglycerate-dependent phosphoglycerate mutase

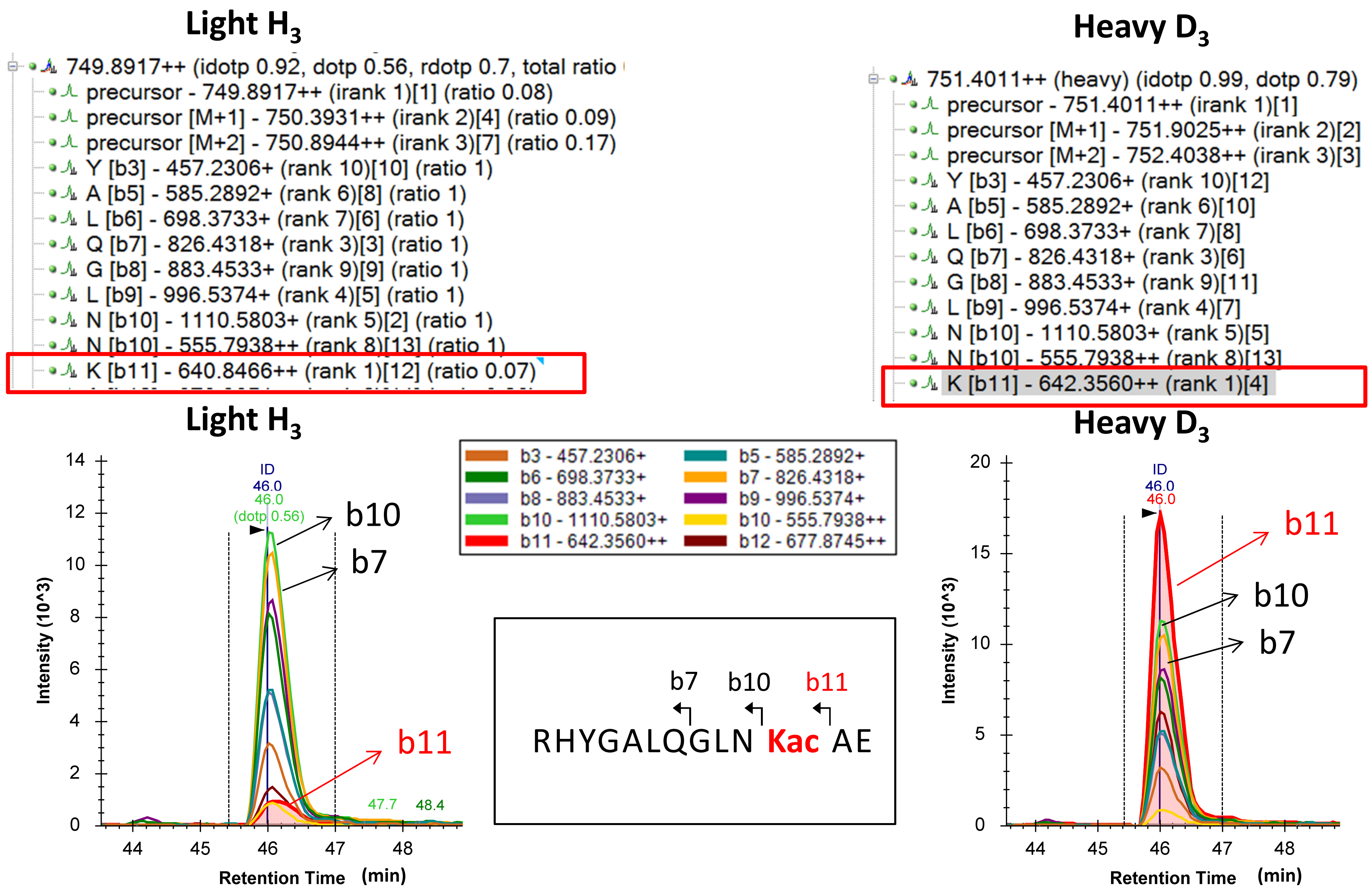

Supplemental Figure 3

(b) GPMA, Display of Glucose-dependent Kac sites

K-100 is the 2-phospho-D-glycerate binding site

GPMA: 2,3-bisphosphoglycerate-dependent phosphoglycerate mutase

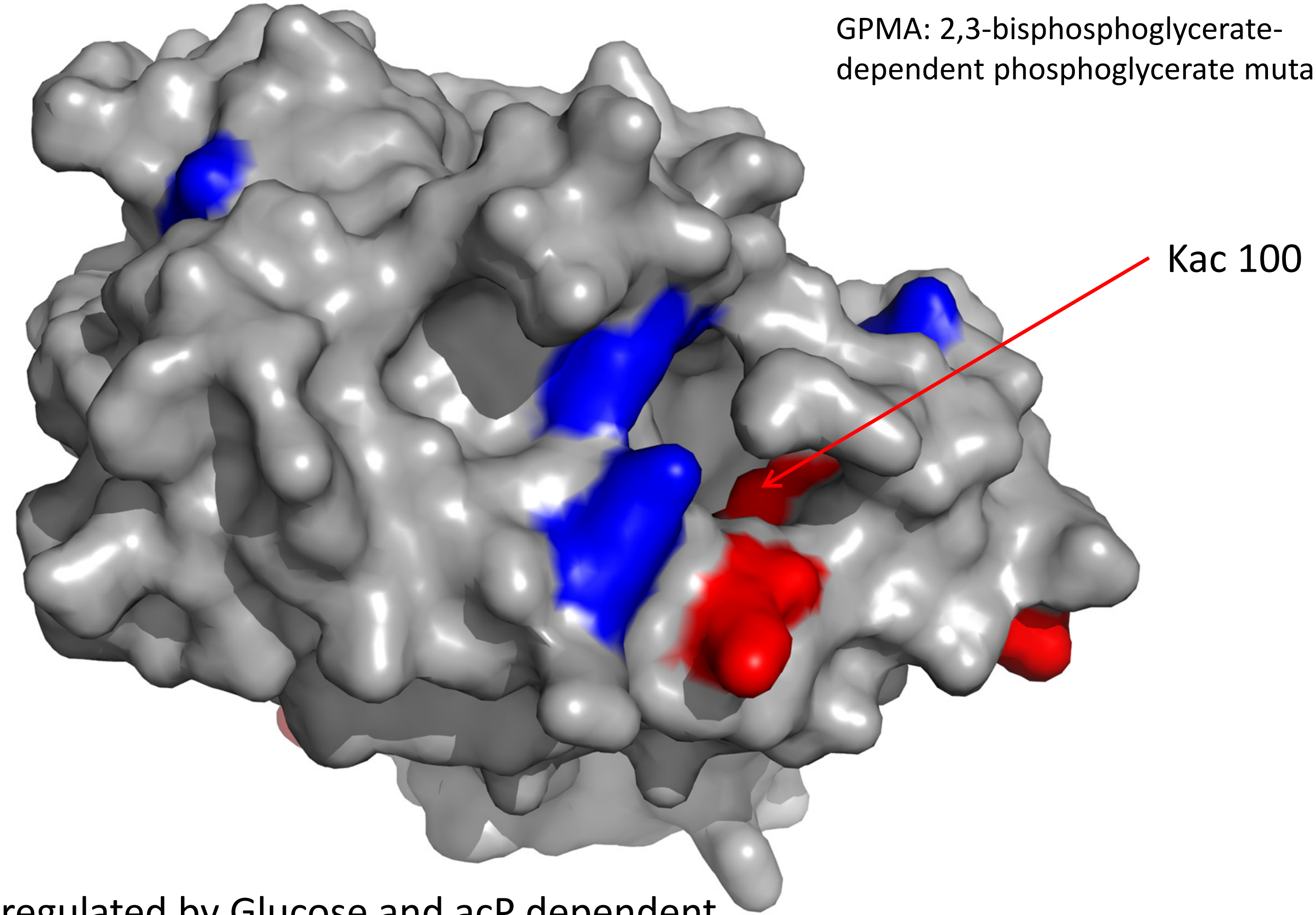

Red: Kac sites regulated by Glucose and acP dependent
Blue: Kac sites regulated by Glucose

Supplemental Figure 3

(c) GPMA, Kac-100, E. coli growth with no glucose supplement

PRM Rep 1

E.RHYGALQGLNKAE.T [89, 101]
749.8917++ (idotp 0.59, dotp 0.72, rdotp 0.45, total ratio
precursor - 749.8917++ (irank 1)[3] (ratio 0.002)
precursor [M+1] - 750.3931++ (irank 2)[2] (ratio 0.004)
precursor [M+2] - 750.8944++ (irank 3)[1] (ratio 0.05)
K [y3] - 389.2031+ (rank 9)[10] (ratio 0.002)
A [b5] - 585.2892+ (rank 8)[9] (ratio 0.002)
L [b6] - 698.3733+ (rank 6)[7] (ratio 0.003)
Q [b7] - 826.4318+ (rank 3)[4] (ratio 0.002)
G [b8] - 883.4533+ (rank 7)[11] (ratio 0.002)
L [b9] - 996.5374+ (rank 5)[8] (ratio 0.002)
N [b10] - 1110.5803+ (rank 4)[6] (ratio 0.002)
K [b11] - 1280.6858+ (rank 1)[12] (ratio 0.002)
K [b11] - 640.8466++ (rank 10)[5] (ratio 0.001)

PRM Rep 2

E.RHYGALQGLNKAE.T [89, 101]
749.8917++ (idotp 0.53, dotp 0.7, rdotp 0.39, total ratio 0
precursor - 749.8917++ (irank 1)[3] (ratio 0.001)
precursor [M+1] - 750.3931++ (irank 2)[2] (ratio 0.005)
precursor [M+2] - 750.8944++ (irank 3)[1] (ratio 0.06)
K [y3] - 389.2031+ (rank 9)[11] (ratio 0.001)
A [b5] - 585.2892+ (rank 8)[9] (ratio 0.002)
L [b6] - 698.3733+ (rank 6)[8] (ratio 0.002)
Q [b7] - 826.4318+ (rank 3)[6] (ratio 0.002)
G [b8] - 883.4533+ (rank 7)[10] (ratio 0.003)
L [b9] - 996.5374+ (rank 5)[5] (ratio 0.003)
N [b10] - 1110.5803+ (rank 4)[7] (ratio 0.002)
K [b11] - 1280.6858+ (rank 1)[12] (ratio 0.001)
K [b11] - 640.8466++ (rank 10)[4] (ratio 0.001)
[illegible]

SWATH (precursor *m/z* 749.89 $^{2+}$)

E.RHYGALQGLNKAE.T [89, 101]
749.8917++ (idotp 0.58, dotp 0.61, rdotp 0.18, total ratio
precursor - 749.8917++ (irank 1)[9] (ratio 0.002)
precursor [M+1] - 750.3931++ (irank 2)[8] (ratio 0.01)
precursor [M+2] - 750.8944++ (irank 3)[7] (ratio 0.07)
K [y3] - 389.2031+ (rank 9)[11] (ratio 0.01)
A [b5] - 585.2892+ (rank 8)[5] (ratio 1)
L [b6] - 698.3733+ (rank 6)[4] (ratio 1)
Q [b7] - 826.4318+ (rank 3)[2] (ratio 1)
G [b8] - 883.4533+ (rank 7)[6] (ratio 1)
L [b9] - 996.5374+ (rank 5)[3] (ratio 1)
N [b10] - 1110.5803+ (rank 4)[1] (ratio 1)
K [b11] - 1280.6858+ (rank 1)[12] (ratio 0.01)
K [b11] - 640.8466++ (rank 10)[10] (ratio 0.01)

SWATH (precursor *m/z* 500.26 $^{3+}$)

500.2636+++ (idotp 0.62, dotp 0.79, rdotp 0.2, total ratio
precursor - 500.2636+++ (irank 1)[11] (ratio 0.01)
precursor [M+1] - 500.5978+++ (irank 2)[12] (ratio 0.01
precursor [M+2] - 500.9320+++ (irank 3)[6] (ratio 0.13)
G [b4] - 514.2521+ (rank 10)[10] (ratio 1)
A [b5] - 585.2892+ (rank 3)[1] (ratio 1)
L [b6] - 698.3733+ (rank 1)[2] (ratio 1)
Q [b7] - 826.4318+ (rank 8)[7] (ratio 1)
G [b8] - 883.4533+ (rank 2)[5] (ratio 1)
L [b9] - 996.5374+ (rank 9)[9] (ratio 1)
N [b10] - 1110.5803+ (rank 6)[8] (ratio 1)
G [b8] - 442.2303++ (rank 7)[3] (ratio 1)
N [b10] - 555.7938++ (rank 5)[4] (ratio 1)
K [b11] - 640.8466++ (rank 4)[13] (ratio 0.004)

Supplemental Figure 3

(d) GPMA, Kac-100, E. coli growth with high glucose supplement

PRM Rep 1

E.RHYGALQGLNKAE.T [89, 101]
- 749.8917++ (idotp 0.94, dotp 0.66, rdotp 0.98, total ratio
  - precursor - 749.8917++ (irank 1)[1] (ratio 0.1)
  - precursor [M+1] - 750.3931++ (irank 2)[2] (ratio 0.11)
  - precursor [M+2] - 750.8944++ (irank 3)[3] (ratio 0.18)
  - K [y3] - 389.2031+ (rank 9)[11] (ratio 0.09)
  - A [b5] - 585.2892+ (rank 8)[9] (ratio 0.09)
  - L [b6] - 698.3733+ (rank 6)[8] (ratio 0.08)
  - Q [b7] - 826.4318+ (rank 3)[5] (ratio 0.1)
  - G [b8] - 883.4533+ (rank 7)[10] (ratio 0.09)
  - L [b9] - 996.5374+ (rank 5)[6] (ratio 0.1)
  - N [b10] - 1110.5803+ (rank 4)[7] (ratio 0.1)
  - K [b11] - 1280.6858+ (rank 1)[12] (ratio 0.1)
  - K [b11] - 640.8466++ (rank 10)[4] (ratio 0.09)

PRM Rep 2

E.RHYGALQGLNKAE.T [89, 101]
- 749.8917++ (idotp 0.94, dotp 0.66, rdotp 0.98, total rati
  - precursor - 749.8917++ (irank 1)[1] (ratio 0.1)
  - precursor [M+1] - 750.3931++ (irank 2)[2] (ratio 0.11)
  - precursor [M+2] - 750.8944++ (irank 3)[3] (ratio 0.18)
  - K [y3] - 389.2031+ (rank 9)[11] (ratio 0.08)
  - A [b5] - 585.2892+ (rank 8)[10] (ratio 0.08)
  - L [b6] - 698.3733+ (rank 6)[8] (ratio 0.08)
  - Q [b7] - 826.4318+ (rank 3)[5] (ratio 0.09)
  - G [b8] - 883.4533+ (rank 7)[9] (ratio 0.1)
  - L [b9] - 996.5374+ (rank 5)[7] (ratio 0.09)
  - N [b10] - 1110.5803+ (rank 4)[6] (ratio 0.1)
  - K [b11] - 1280.6858+ (rank 1)[12] (ratio 0.1)
  - K [b11] - 640.8466++ (rank 10)[4] (ratio 0.09)

SWATH (precursor *m/z* 749.89 $^{2+}$)

E.RHYGALQGLNKAE.T [89, 101]
- 749.8917++ (idotp 0.94, dotp 0.66, rdotp 0.78, total ratio
  - precursor - 749.8917++ (irank 1)[1] (ratio 0.1)
  - precursor [M+1] - 750.3931++ (irank 2)[2] (ratio 0.11)
  - precursor [M+2] - 750.8944++ (irank 3)[5] (ratio 0.18)
  - K [y3] - 389.2031+ (rank 9)[11] (ratio 0.1)
  - A [b5] - 585.2892+ (rank 8)[9] (ratio 1)
  - L [b6] - 698.3733+ (rank 6)[7] (ratio 1)
  - Q [b7] - 826.4318+ (rank 3)[4] (ratio 1)
  - G [b8] - 883.4533+ (rank 7)[8] (ratio 1)
  - L [b9] - 996.5374+ (rank 5)[6] (ratio 1)
  - N [b10] - 1110.5803+ (rank 4)[3] (ratio 1)
  - K [b11] - 1280.6858+ (rank 1)[12] (ratio 0.13)
  - K [b11] - 640.8466++ (rank 10)[10] (ratio 0.07)

SWATH (precursor *m/z* 500.26 $^{3+}$)

- 500.2636+++ (idotp 0.93, dotp 0.87, rdotp 0.75, total rati
  - precursor - 500.2636+++ (irank 1)[1] (ratio 0.1)
  - precursor [M+1] - 500.5978+++ (irank 2)[3] (ratio 0.12)
  - precursor [M+2] - 500.9320+++ (irank 3)[4] (ratio 0.27)
  - G [b4] - 514.2521+ (rank 10)[12] (ratio 1)
  - A [b5] - 585.2892+ (rank 3)[2] (ratio 1)
  - L [b6] - 698.3733+ (rank 1)[5] (ratio 1)
  - Q [b7] - 826.4318+ (rank 8)[9] (ratio 1)
  - G [b8] - 883.4533+ (rank 2)[8] (ratio 1)
  - L [b9] - 996.5374+ (rank 9)[11] (ratio 1)
  - N [b10] - 1110.5803+ (rank 6)[10] (ratio 1)
  - G [b8] - 442.2303++ (rank 7)[6] (ratio 1)
  - N [b10] - 555.7938++ (rank 5)[7] (ratio 1)
  - K [b11] - 640.8466++ (rank 4)[13] (ratio 0.1)

Supplementary Figure S4.

Stoichiometry examples. **(a)** Three independent biological replicates of *E. coli* grown with high glucose were prepared and processed, and a selected subset of sites is shown for which acetylation occupancy was calculated for acetylation sites from proteins ClpB, DnaK and LRP using this new SWATH MS2 quantification method. **(b)** Occupancy discrepancy between MS1 & MS2 measurements.

Supplementary Figure S4A: MS2 SWATH *E. coli* -- Kac occupancy

Chaperone protein ClpB

Gene Name	Acetyl Site Kac	expmt	MS2 SWATH % occupancy L/(L+H) in %
clpB	176	CR2	0.2
clpB	176	DC3	0.4
clpB	176	DC2	0.1
clpB	250	CR2	0.15
clpB	250	DC3	0.12
clpB	250	DC2	0.17
clpB	260	DC3	0.1
clpB	260	DC2	0.1
clpB	335	DC3	0.6
clpB	335	DC2	0.6
clpB	354	CR2	0.65
clpB	354	DC3	0.06
clpB	354	DC2	0.3
clpB	842	CR2	0.67
clpB	842	DC3	0.39
clpB	842	DC2	0.4

- Data for 2-3 bio replicates (CR2, DC3, DC2)

Chaperone protein DnaK

Gene Name	Acetyl Site Kac	expmt	MS2 SWATH % occupancy L/(L+H) in %
dnaK	155	CR2	5.89
dnaK	155	DC3	4.82
dnaK	155	DC2	5.07
dnaK	514	CR2	0.99
dnaK	514	DC2	0.24
dnaK	514	DC3	0.08
dnaK	528	CR2	0.56
dnaK	528	DC3	0.31
dnaK	528	DC2	0.40
dnaK	548	DC3	0.4
dnaK	548	DC2	0.5
dnaK	548	CR2	2
dnaK	597	CR2	1.39
dnaK	597	DC3	2.03
dnaK	597	DC2	2.97
**Leucine-responsive regulatory protein**			
lrp	25	CR2	5.48
lrp	25	DC3	4.62
lrp	25	DC2	4.60

Supplementary Figure S4B. Occupancy discrepancy between MS1 & MS2 measurements

Reproducibility considerations measuring multiple biological replicates

Stoichiometry was determined from 3 biological replicates

SwProt	Protein Description	Gene Name	Acetyl Site	expmt	MS2 SWATH SIGNAL % occupancy L/(L+H) in %	MS1 SIGNAL % occupancy L/(L+H) in %
C4ZXS6	2,3-bisphosphoglycerate-dependent phosphoglycera	gpmA	146	CR2	1.42	1.38
C4ZXS6	2,3-bisphosphoglycerate-dependent phosphoglycera	gpmA	146	DC3	0.47	17.66
C4ZXS6	2,3-bisphosphoglycerate-dependent phosphoglycera	gpmA	146	DC2	0.61	8.76
C4ZYK2	Autonomous glycyl radical cofactor	grcA	48	CR2	1.66	10.24
C4ZYK2	Autonomous glycyl radical cofactor	grcA	48	DC3	1.95	7.34
C4ZYK2	Autonomous glycyl radical cofactor	grcA	48	DC2	3.23	39.1

- ✓ **MS2** measurements show very good reproducibility between biological replicates per Kac site
- ✓ **MS1** measurements are more inconsistent between biological replicates per Kac site

Supplementary Figure S5.

Assessing potential (MS1) interferences during stoichiometry analysis and reducing complexity of samples by offline fractionation. **(a**, **b)** MS1 interferences present in unseparated peptide samples from *E. coli* were removed after basic pH reversed-phase fractionation. **(a)** XICs for peptide YSVIQT**Ksucc**EPLDRE from protein GALF (P0AAB6) with doubly charged heavy precursor M at *m/z* 841.43. **(b)** XICs for peptide VMV**Ksucc**GPGPGRE from protein RS11 (B1X6E9) with doubly charged heavy precursor M at *m/z* 623.82. **(c,d)** MS2 interferences present in unseparated peptide samples from *E. coli* were removed in by basic pH reversed-phase fractionation. **(c)** XICs for peptide ATSDYDRE**Ksucc**LQE from protein CH60 (C5A1D5) with doubly charged heavy precursor M at *m/z* 779.86. **(d)** XICs for peptide FI**Ksucc**PEDVSAE from protein EFTS (C4ZRR2) with doubly charged, heavy precursor M at *m/z* 619.81.

Supplemental Figure 5

MS1 Interferences

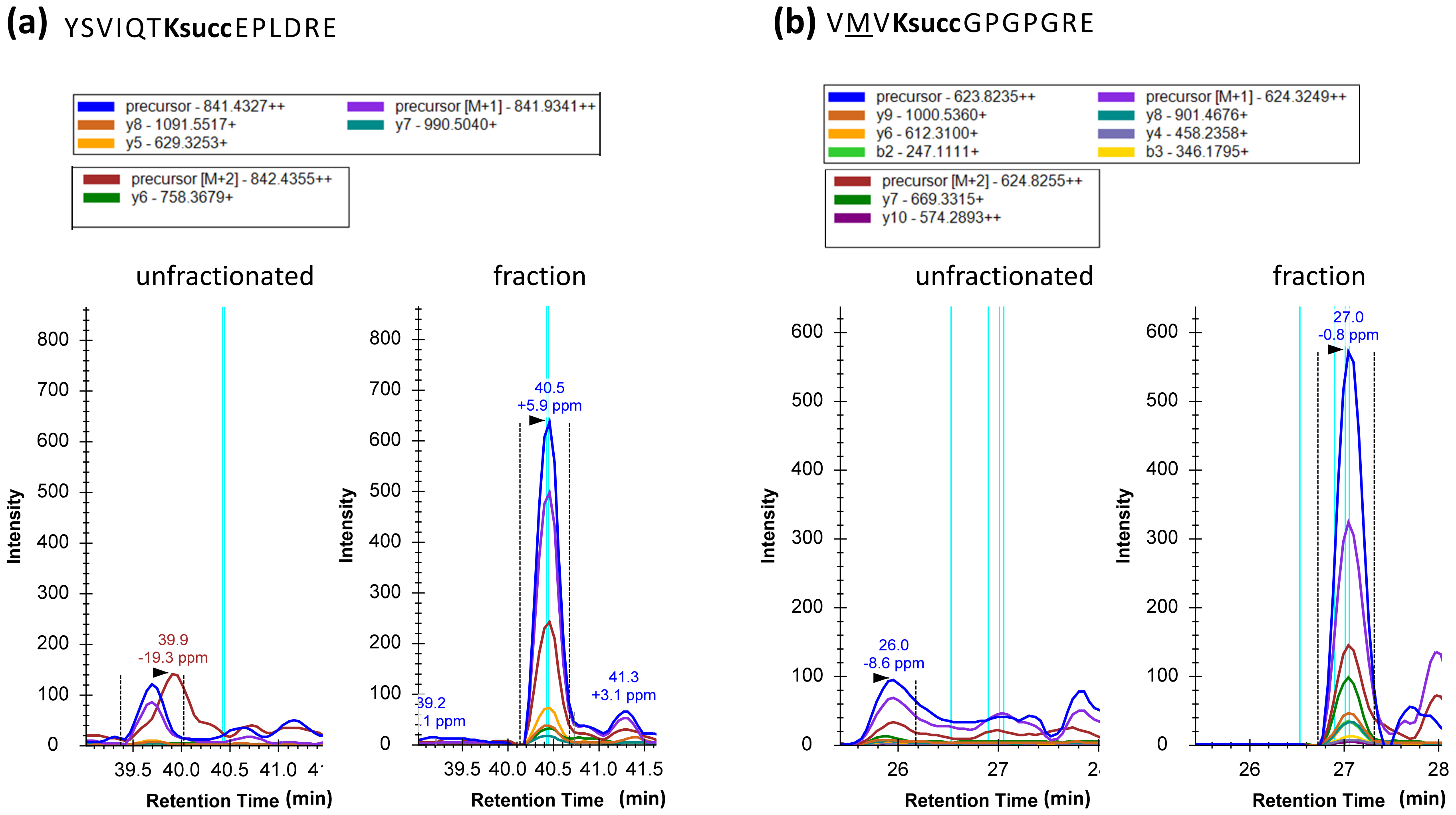

Supplemental Figure 5

MS2 Interferences

(c) ATSDYDRE**Ksucc**LQE

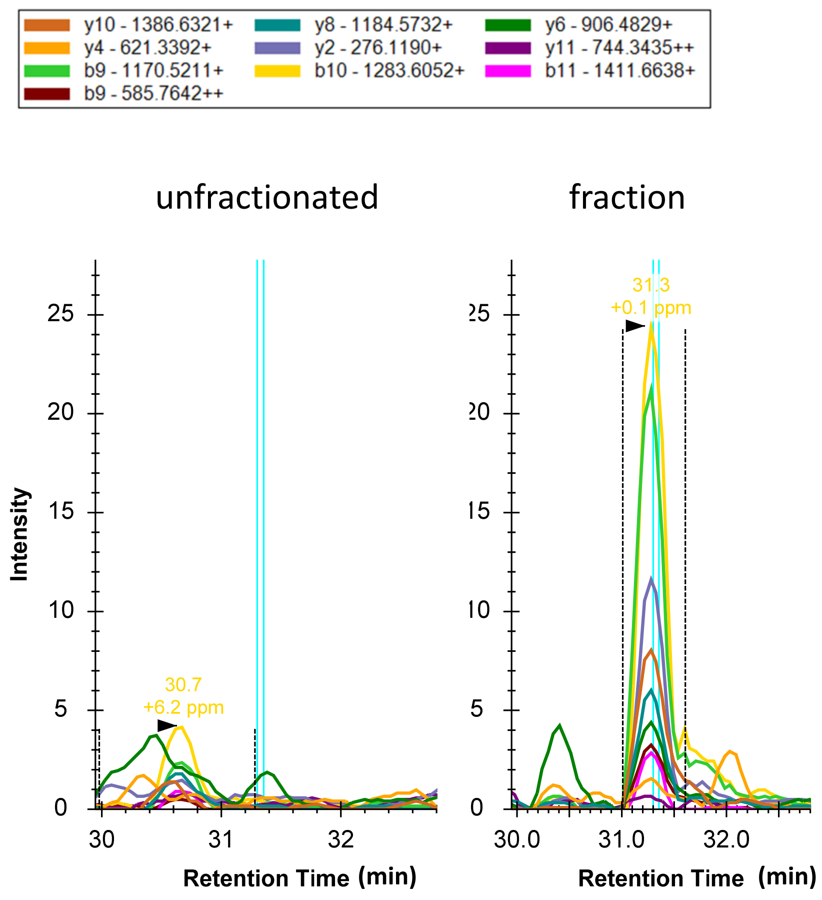

(d) FI**Ksucc**PEDVSAE

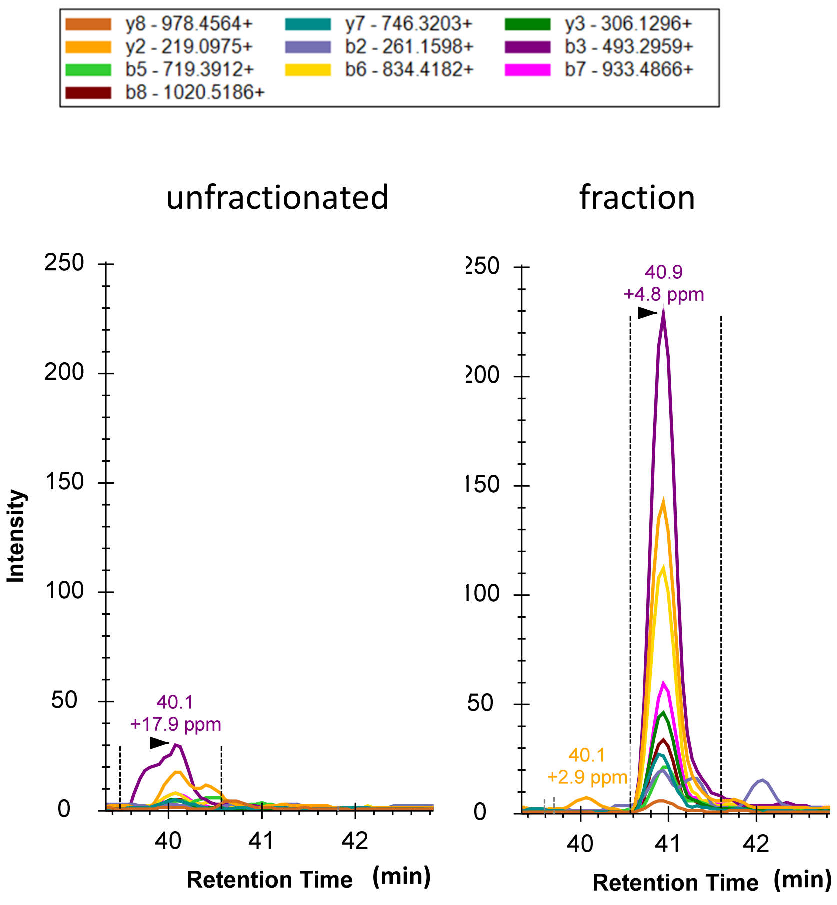

Supplementary Figure S6.

The role of sample fractionation for stoichiometry analysis. Succinylation site occupancy for all single-lysine peptides measured from the *E. coli* experiment comparing the distribution of values determined using combinations of MS1 and MS2 based calculations. Unseparated samples showed slightly higher stoichiometry measurements compared to basic reversed-phase fractions (after fractionation of whole lysate), suggesting that fractionation reduces possible interferences.

Supplemental Figure 6

Succinylation site occupancy: *E. coli* lysate – analysis of whole lysate (unseparated) and fractions (basic reversed-phase fractionation)

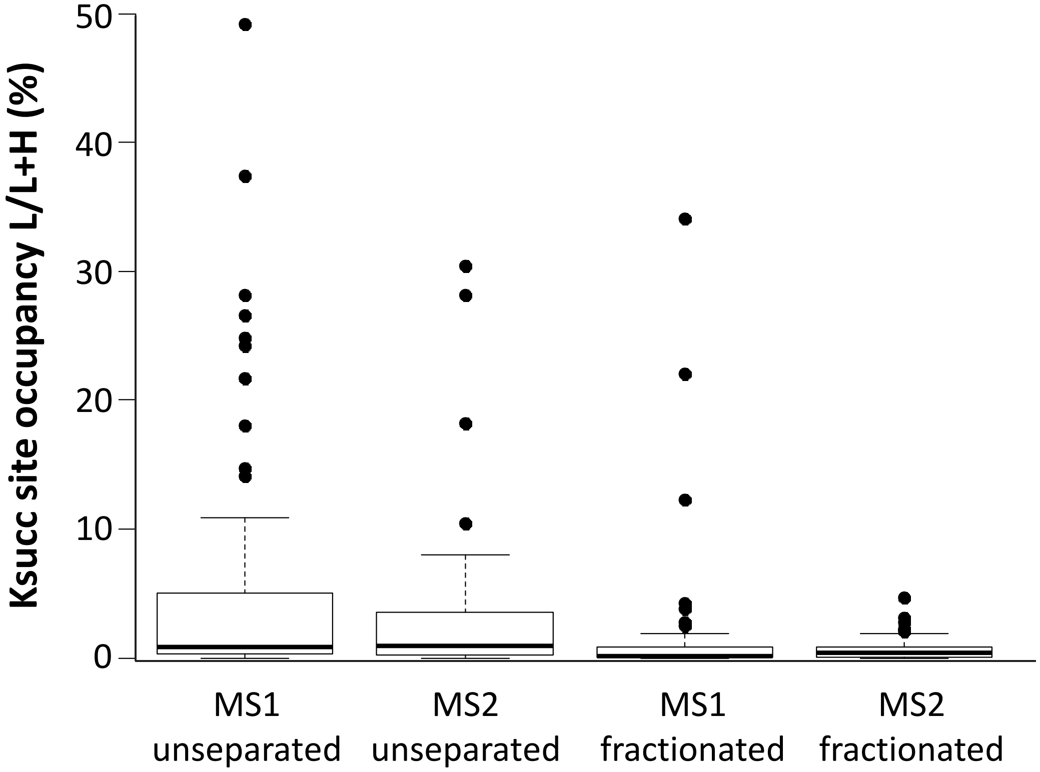

Supplementary Figure S7.

Example set-up of peptides containing two acyl groups in Skyline's modification interface. The Edit Modification table in Skyline allows set up of structural modifications, i.e. acetyl on Lys residues, as well as isotope modifications. This allows population of the tree with all possible combinations of Light/Light, Heavy/Heavy, Light/Heavy and Heavy/Light peptide species that are needed to visualize endogenously and exogenously acetylated peptides.

Supplemental Figure 7a

Skyline setup for multiple Lysine residues per peptide quantified

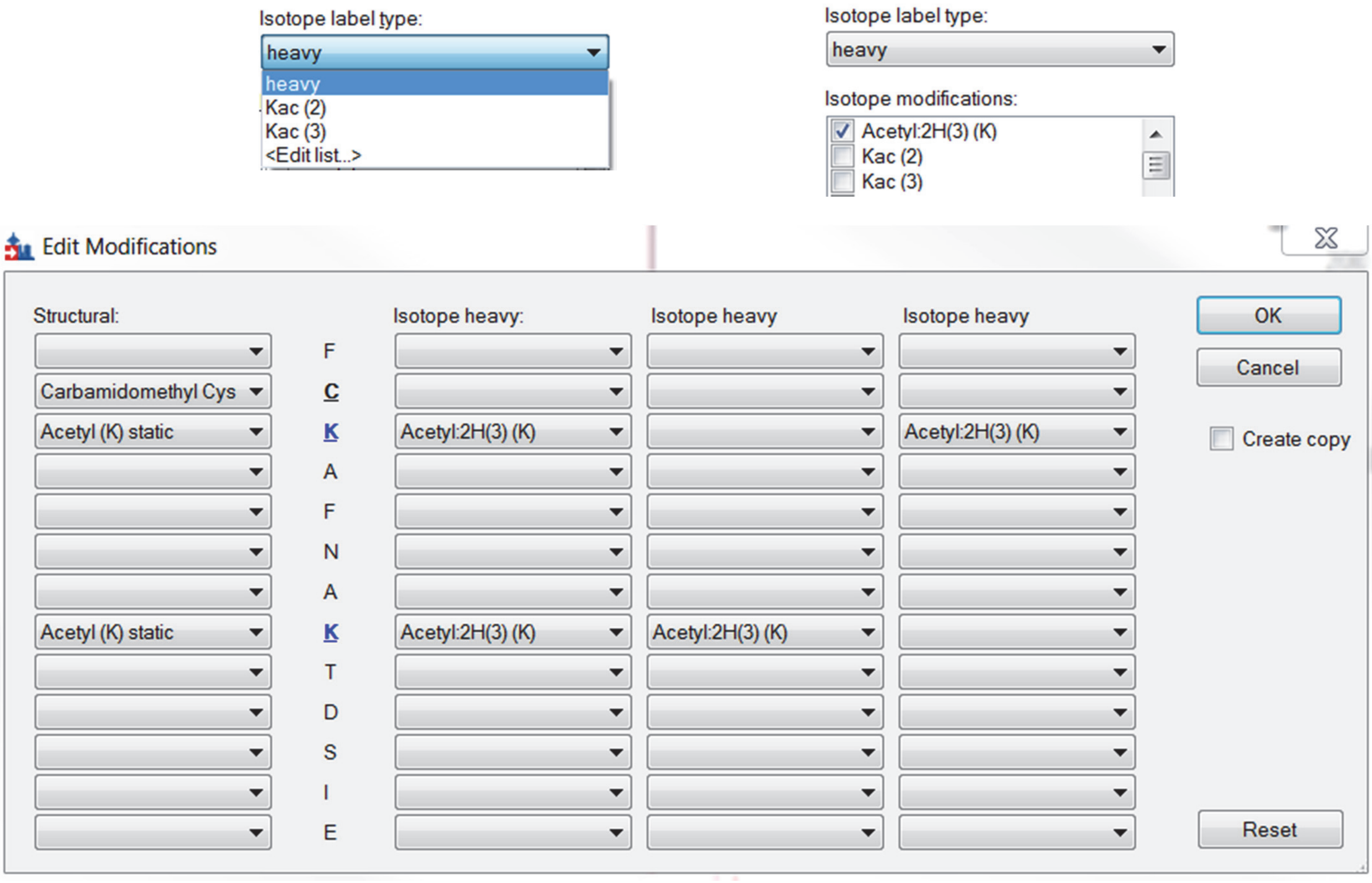

Supplemental Figure 7b

Untargeted Computational Workflow to Determine Stoichiometry

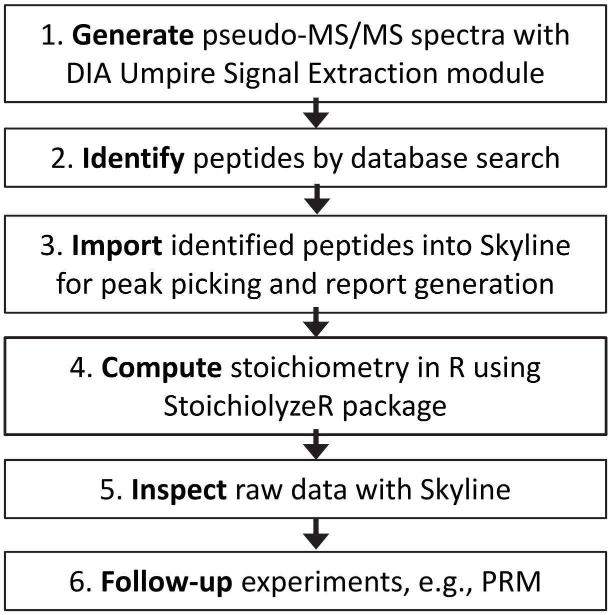

Computation of lysine stoichiometry from a peptide containing two lysine residues. Generally, for any peptide sequence containing two lysine residues ordered from n-terminal to c-terminal, k_1 and k_2, the acyl stoichiometry of k_1 can be computed using b-ions that contain only the first lysine. If *m* is the position of the first lysine in the peptide, *n* is the position of the second lysine, and *p* is the length of the peptide, the acyl stoichiometry of k_1 is calculated using light, bL, and heavy, bH, areas of any b-ion number *i*, where *i* is between *i=m* and *i=n-1,* according to the equation:

$$stoichiometry_{k_1} = \frac{(bL_i)}{(bL_i) + (bH_i)}$$

Similarly, the acyl stoichiometry of k_2 can be computed using any y-ion areas that contain only the second lysine with lengths between *i=p-n* and *i=p-m-1* according to the equation:

$$stoichiometry_{k_2} = \frac{(yL_i)}{(yL_i) + (yH_i)}$$

For example, if we have the sequence AFCK_1RNFK_2GE, the stoichiometry of K_1 can be computed from light and heavy areas of any b-ion between 4 and 7, for example, using the equation:

$$stoichiometry_{K_1} = \frac{(bL5)}{(bL5) + (bH5)}$$

Similarly, the stoichiometry of K_2 can be computed from the light and heavy areas of any y-ion between 3 and 6, for example, using the equation:

$$stoichiometry_{K_2} = \frac{(yL3)}{(yL3) + (yH3)}$$

Supplementary Figure S8. Distribution of acylation stoichiometry measurements as determined from *E. coli* samples for **(a)** acetylation sites and **(b)** succinylation sites.

Supplemental Figure 8a

Distribution of monitored acetylation stoichiometries for 216 monitored lysine acetylation sites

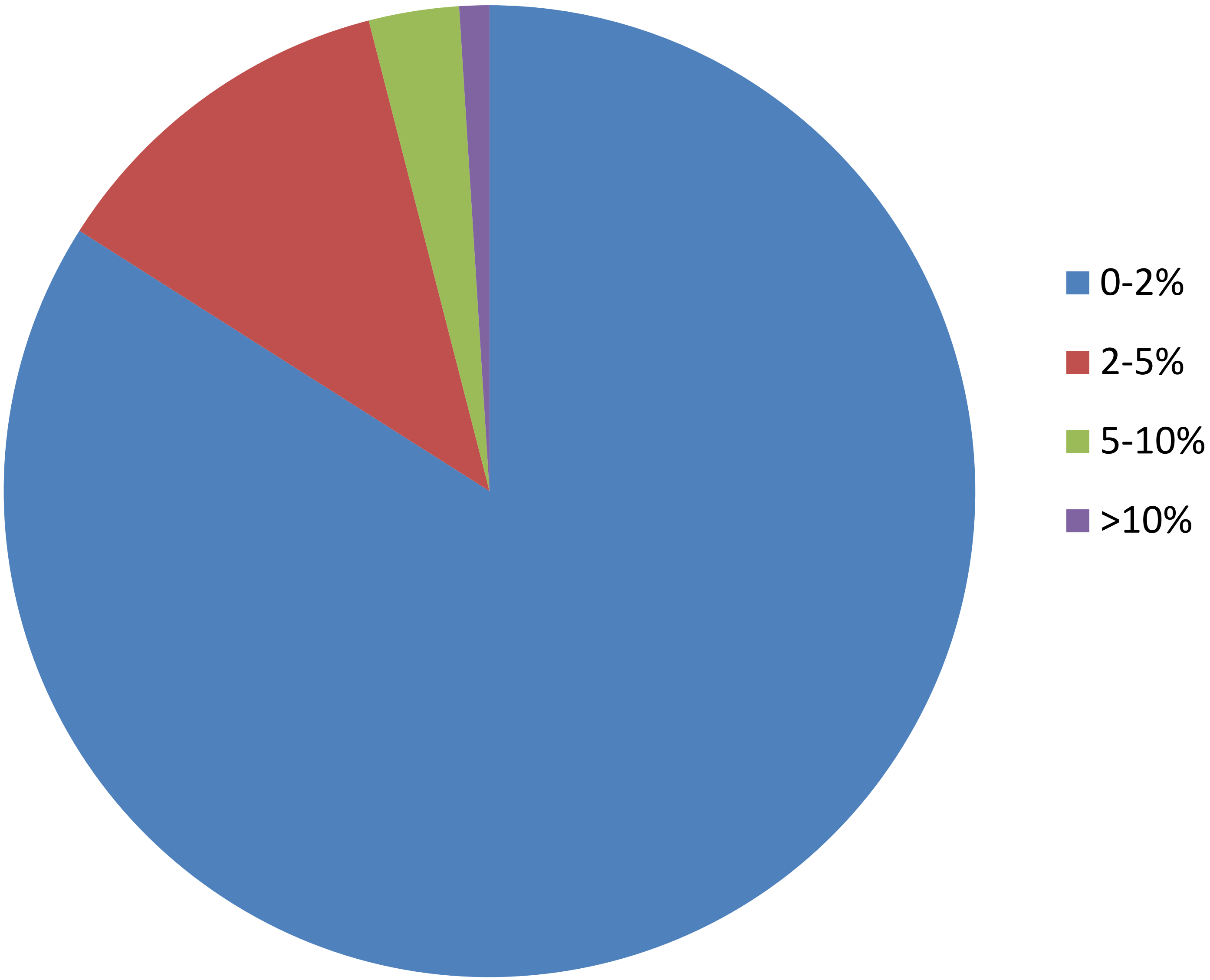

Supplemental Figure 8b

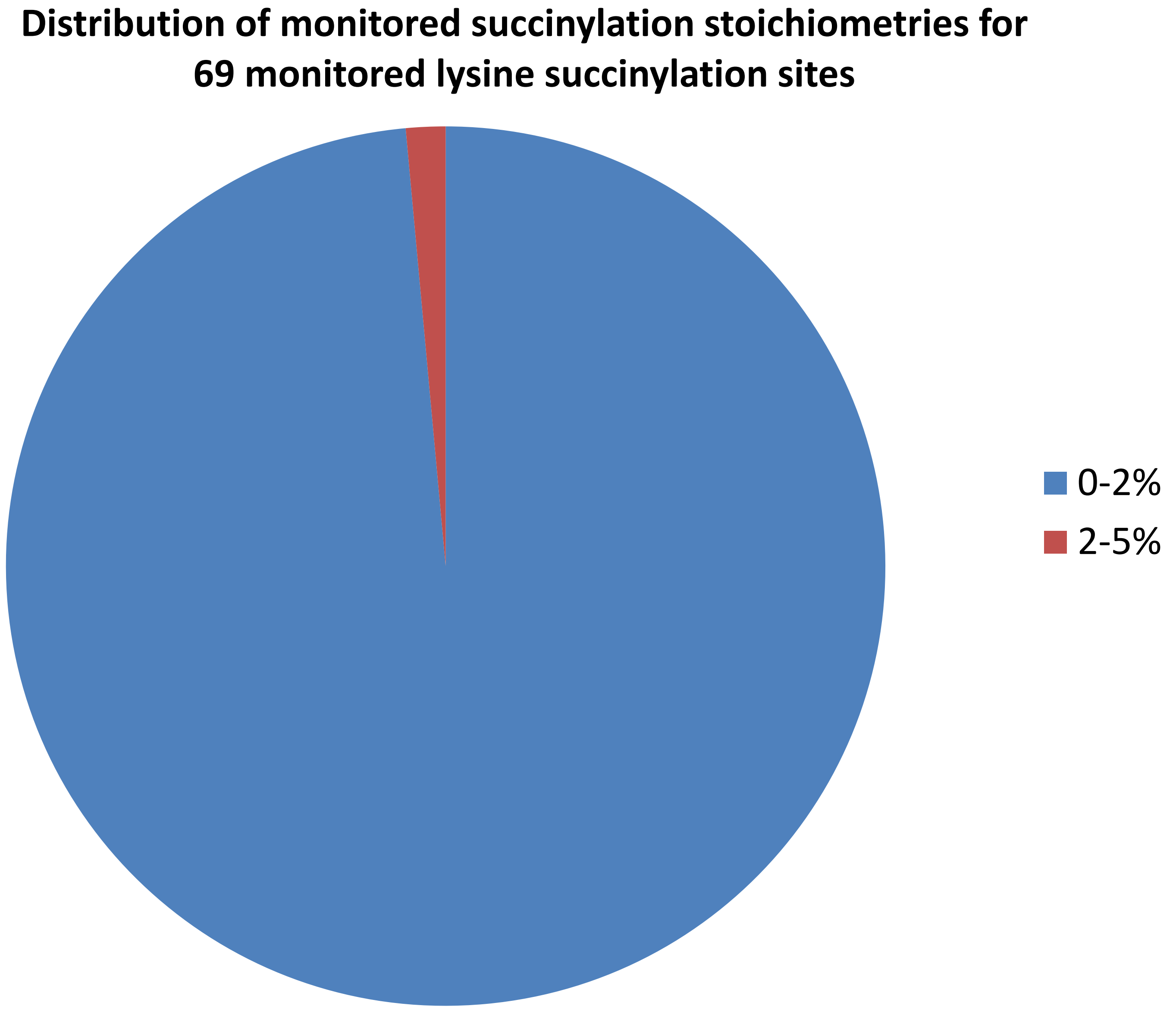

Supplement: Supplementary file 1 — (PDF 6.37 mb) [file 13361_2016_1476_MOESM1_ESM.pdf]
